# Multiplexed DNA-functionalized graphene sensor with artificial intelligence-based discrimination performance for analyzing chemical vapor compositions

**DOI:** 10.1038/s41378-023-00499-y

**Published:** 2023-03-20

**Authors:** Yun Ji Hwang, Heejin Yu, Gilho Lee, Iman Shackery, Jin Seong, Youngmo Jung, Seung-Hyun Sung, Jongeun Choi, Seong Chan Jun

**Affiliations:** School of Mechanical Engineering, Yonsei University, 50, Yonsei-ro, Seodaemun-gu, Seoul, 03722 Republic of Korea

**Keywords:** Nanosensors, Electronic properties and materials, Computational nanotechnology

## Abstract

This study presents a new technology that can detect and discriminate individual chemical vapors to determine the chemical vapor composition of mixed chemical composition in situ based on a multiplexed DNA-functionalized graphene (MDFG) nanoelectrode without the need to condense the original vapor or target dilution. To the best of our knowledge, our artificial intelligence (AI)-operated arrayed electrodes were capable of identifying the compositions of mixed chemical gases with a mixed ratio in the early stage. This innovative technology comprised an optimized combination of nanodeposited arrayed electrodes and artificial intelligence techniques with advanced sensing capabilities that could operate within biological limits, resulting in the verification of mixed vapor chemical components. Highly selective sensors that are tolerant to high humidity levels provide a target for “breath chemovapor fingerprinting” for the early diagnosis of diseases. The feature selection analysis achieved recognition rates of 99% and above under low-humidity conditions and 98% and above under humid conditions for mixed chemical compositions. The 1D convolutional neural network analysis performed better, discriminating the compositional state of chemical vapor under low- and high-humidity conditions almost perfectly. This study provides a basis for the use of a multiplexed DNA-functionalized graphene gas sensor array and artificial intelligence-based discrimination of chemical vapor compositions in breath analysis applications.

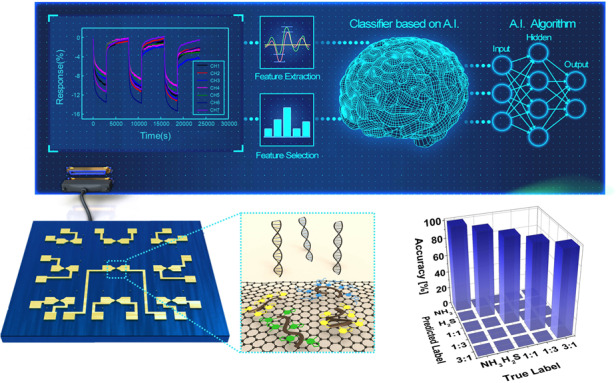

## Introduction

Chemical vapor sensors play a vital role in various applications, such as in medical diagnosis, aerospace, military, environmental monitoring, and industrial production and safety^1^. Various gases are released in significant volumes by industries as well as by the human body. Even small amounts of such gases can negatively affect the human body, organisms, and environment^2–4^. Recently, interest in breath analysis technology has greatly increased, particularly in the field of disease diagnosis, and several studies have been conducted on the detection of complex biomarker gases related to physical conditions and diseases; typical biomarkers include ammonia (NH_3_), hydrogen sulfide (H_2_S), and nitric oxide (NO)^5,6^. NH_3_ is present in the exhaled breath of patients with diseases such as asthma, liver disease, kidney disease, stomach ulcers, and duodenal ulcers. H_2_S is present in the exhaled breath of patients with malodor, lung cancer, pancreatitis, and asthma. NO is present in the exhaled breath of patients with lung cancer and asthma^7–12^. Furthermore, related biomarker detection techniques enable the early detection of diseases and are noninvasive and painless. To enhance their development and determine the target gas content in a mixed gas state, gas species should be characterized in a humid environment (~80% humidity) to simulate the properties of exhaled human breath^13^. Recently, wearable and stretchable gas sensors have received tremendous attention. Lei Zhang et al. reported a highly sensitive wireless gas sensor^14^. In addition, Li Yang et al. reported a novel gas-sensing platform based on stretchable laser-induced graphene, and Yi Ning et al. reported stretchable gas sensors for detecting biomarkers from humans and in exposed environments^15,16^. In addition, research on flexible gas sensors continues today^17,18^.

Currently, several gas sensors are available; among them, solid-state gas sensors offer considerable benefits owing to their low cost, low energy demand, small form factor, and high sensitivity. However, these sensors have certain disadvantages—they may not perform consistently over extended periods of use, and their measurement precision may be restricted^19^. Generally, to determine the sensing performance of a gas sensor, four important parameters are considered: the working temperature, sensitivity, selectivity, and response/recovery time. Thus, researchers have focused on the development of various techniques to enhance or increase the selectivity, reduce the response/recovery time, and decrease the working temperature. Over the past few years, gas sensors based on metal oxides such as ZnO, TiO_2_, and SnO_2_ have been widely used owing to their speed and high sensitivity in gas detection^20^. However, the majority of general gas-sensor metal oxides require high temperatures for their operation, which limits their application in various fields. Consequently, the development of high-efficiency gas sensors remains a challenging task. In addition, such sensors are often disadvantageous because they may not yield uniform measurements, particularly under high-humidity conditions. To overcome this, research is being conducted to minimize the effect of humidity by utilizing a membrane-type structure^21^. Recently, graphene-based sensors have attracted significant attention^22–25^. Such sensors can be operated at room temperature owing to their unique structures and remarkable chemical, electronic, mechanical, optical, and thermal properties^26^. Graphene-based materials can be used to measure sensing characteristics owing to their large specific surface area and desirable electrical characteristics, such as low electrical noise, high electron mobility, and high conductivity under atmospheric conditions. In this study, by utilizing multiplexed DNA-functionalized graphene (MDFG), a large range of biomolecules, chemicals, gases, and vapors were successfully detected^27–33^.

To enhance the gas-sensing performance of graphene-based gas sensors, different techniques, such as oxidization, doping, and decoration of graphene using nanoparticles, have been employed, and the compositions of other materials have been investigated. All these methods were used to enhance the gas-sensing properties of graphene^34–37^. The detection of mixtures of different gases is a prerequisite for the practical applications of gas sensors. Nevertheless, the in situ detection of gas mixtures under high-humidity conditions via a single sensor is a complex task because of the deterioration of the sensor signals^38,39^. This challenge can be overcome by employing a set of corresponding sensors for the simultaneous examination of numerous sensor signals through a normalized model to appropriately detect the constituent gases through the application of a single-stranded DNA-functionalized graphene (ssDNA-FG) gas sensor. An ssDNA-FG gas sensor has an additional ion conduction channel composed of H_3_O+ in the presence of water molecules, which improves the performance of the sensor in high-humidity environments^40^. Therefore, a high-performance gas-sensor array should be configured to resolve these problems. In this study, we constructed a sensor array using graphene, which was functionalized using single-stranded DNA (ssDNA), to change the surface properties of a sensor device. The ssDNA-FG gas sensor can improve the sensor performance, such as the reactivity of the sensor under high-humidity conditions, and can be implemented in breath analyzers^40^. Seven arrays of graphene-based ssDNA gas sensors were constructed on a single chip to detect NO_2_, NO, NH_3_, and H_2_S in an individual gas and a combination of gas mixtures, and our sensor array exhibited a high sensitivity.

A limited number of studies have been conducted on the discriminative analysis of gas species in gas mixtures of exhaled breath in situ. Furthermore, sufficient information regarding the detection of mixed gases has not been established, and the recognition rate for mixed gases under high-humidity conditions is considerably low. In this study, an artificial intelligence algorithm was employed for the discrimination of gas mixtures. This approach was applied for the discrimination of gas species via the Boruta algorithm, which is a feature selection machine learning algorithm. For the performance analysis of feature selection, we also conducted gas species discrimination using a support vector machine (SVM) classification algorithm. According to the obtained results, the algorithm performed satisfactorily under dry conditions, and the gas mixture was effectively classified under high-humidity conditions, thereby verifying the applicability of the MDFG for breath analysis. Through these analyses, we successfully enhanced the classification accuracy; an analysis accuracy of up to 98% was achieved. In addition, in this study, the discrimination of gas species in mixed gases was performed with a deep learning model designed based on a 1D convolutional neural network (CNN), which is widely used for processing time series data^41^. By achieving faultless discrimination of the mixed gas composition at random under low- and high-humidity conditions, we demonstrated that the algorithm developed in this study efficiently classified the species within a gas mixture and confirmed that it outperformed machine learning. We expect that the evolution of highly sensitive sensor arrays and strategies involving the utilization of artificial intelligence will contribute to the discrimination of gas species in chemical vapor compositions that are uncommon in the real world.

## Results

**Fig. 1:**
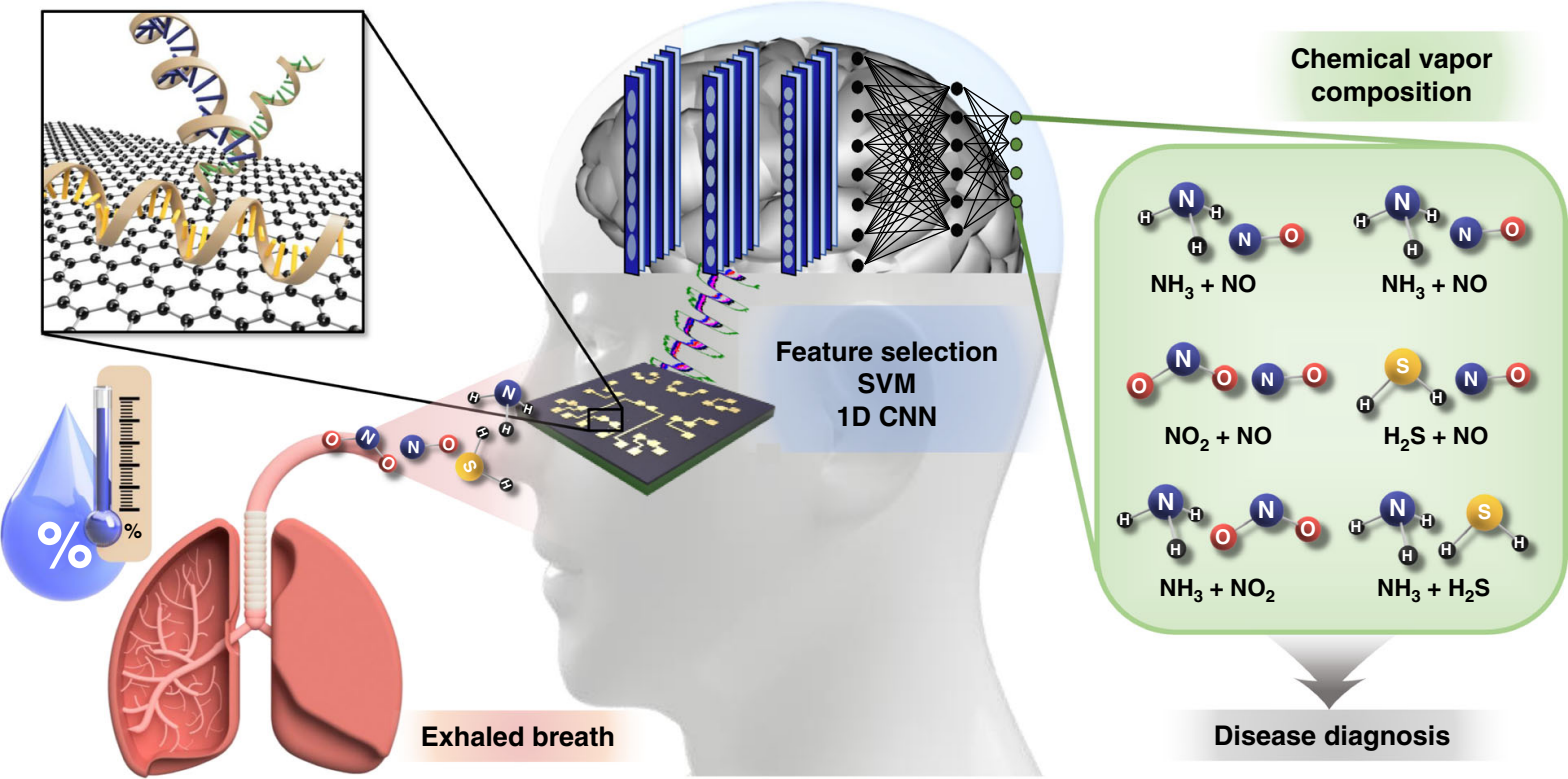
Schematic diagram of the human exhaled gas diagnostic process. Schematic diagram of the process of diagnosing diseases by sensing constituent chemical vapors of exhaled breath through a multiplexed DNA-functionalized graphene sensor and identifying gases through artificial intelligence. NH_3_, NO, NO_2_, and H_2_S molecules that exist individually or in mixed states in exhaled human breath (under conditions of considerable humidity) are detected by the gas sensor array to which the DNA sequence is applied; the presence of these molecules is conveyed through an electrical signal. Their identification is achieved through artificial intelligence via feature selection, support vector machine (SVM), and 1D CNN for the diagnosis of lung and liver diseases. Adapted with permission^42,43^

The process of diagnosing lung and liver diseases using exhaled breath is illustrated in Fig. 1^42,43^. The chemical vapor mixture under humid conditions is released from the exhaled breath of humans. We injected NH_3_, NO, NO_2_, and H_2_S under low- and high-humidity conditions. These chemical mixtures were verified by our graphene-based ssDNA sensor array and output as an electrical signal, which was used to identify the mixed state of the chemical vapor composition through artificial intelligence via feature selection, SVM, and 1D CNN. Based on these results, diseases such as asthma, liver diseases, kidney diseases, stomach ulcers, and duodenal ulcers can be diagnosed^7–12^.

### Design and fabrication of a gas sensor array

**Fig. 2:**
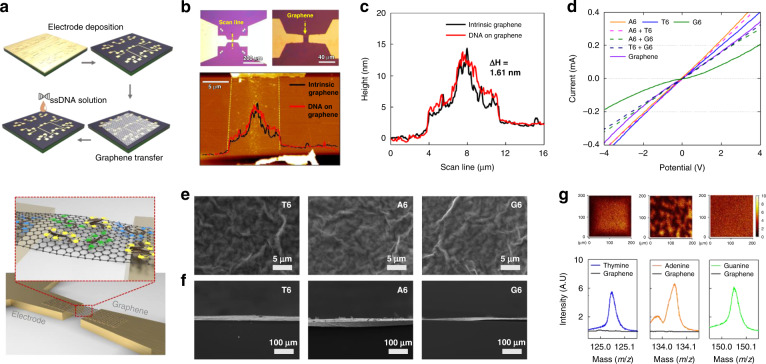
Sensor device and properties. **a** Schematic of the fabrication process of the graphene-DNA gas sensor array and conductive channel. **b** Optical and AFM images of the gas-sensing channel. **c** The height difference between AFM measurements before and after ssDNA adsorption was 1.61 nm. G peak (**d**) of Raman shift. **e** Top-view SEM images of graphene using functionalized thymine, adenine, and guanine sequences and cross-sectional-view SEM images (**f**) of graphene using functionalized thymine, adenine, and guanine sequences. **g** Mapping TOF-SIMS image of graphene using functionalized thymine, adenine, and guanine sequences, showing the magnitude spectral peaks at masses of 125 (thymine), 134 (adenine), and 150 m z−1 (guanine)

The sensor array was fabricated using monolayer graphene. Prior to fabrication, graphene should be functionalized by employing one of the following characteristics: nanoparticles, DNA, and organic materials^34,36^. First, we functionalized graphene using ssDNA and subsequently fabricated a sensor array, which exhibited increased reactivity to the gas through the implementation of DNA. In addition, this method of preparation offers certain advantages, such as simple synthesis and sensor array production based on the DNA sequence. Six graphene-DNA sensors and one pristine graphene sensor were fabricated; the DNA sequences AAA-AAA (A6), TTT-TTT (T6), and GGG-GGG (G6) were utilized for this purpose. Three sensors were used for the A6, T6, and G6 sequences, and three sensors were thereafter constructed by utilizing the A6T6, A6G6, and T6G6 sequences. The sensor fabrication process is illustrated in Fig. 2a.

**Fig. 3:**
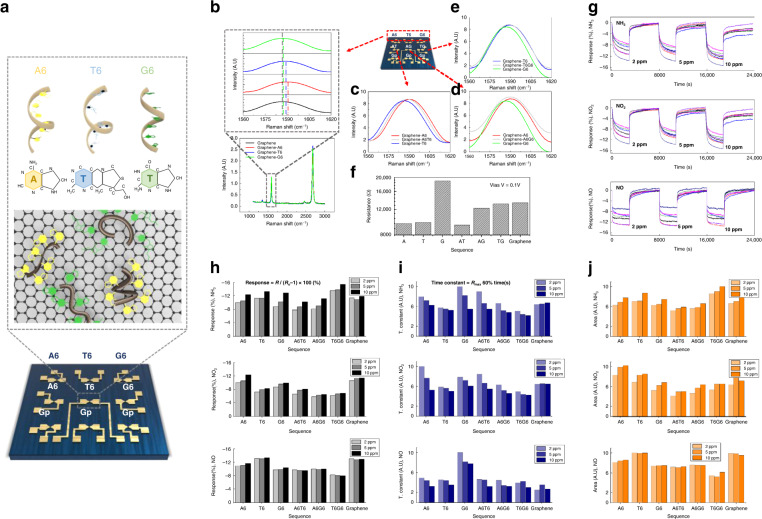
Sensing characteristics by gas concentrations. **a** DNA sequence and placement applied in the graphene-DNA gas sensor array for sensing gases under low- and high-humidity conditions and G-peak shifts of Raman spectra according to DNA sequences of **b** A6, T6, G6, **c** A6T6, **d** A6G6, and **e** T6G6. **f** Resistance according to the bias (V) of the sensor array channel. The **g** response, **h** maximum reactivity, **i** time constant, and **j** area used as the response toward NH_3_, NO_2_, and NO gases at 2, 5, and 10 ppm, respectively

Graphene-ssDNA-based gas sensor arrays were fabricated through the following three steps: (i) electrode deposition, (ii) graphene deposition and patterning, and (iii) ssDNA functionalization. The sensor array was fabricated using SiO_2_ (1 μm)/Si (500 μm) wafers through a mass production process. A 100-nm-thick electrode was molded via photolithography and Au sputter deposition, as depicted in Fig. 2a. The graphene deposited through chemical vapor deposition was patterned between the Au electrodes via photolithography and an O_2_ plasma process. After annealing, the ssDNA was functionalized using droplets under 100% relative humidity for 3 h. The fabricated sensor arrays were 15 mm × 15 mm in size. The formation of monolayer graphene was confirmed through Raman analysis before and after annealing, and the peak intensity ratio of 2D to G was 1.73 (I2D/IG) (refer to Figure S1). In addition, optical and atomic force microscopy (AFM) images are presented in Fig. 2b. Graphene binds to s-DNA through π–π stacking, and after ssDNA binding, the adsorption of ssDNA onto graphene based on the differences in height was confirmed through AFM measurements (refer to Fig. 2c)^44,45^. The current–voltage (I–V) characteristics of the changed sensor array, which confirmed the functionality of the ssDNA in the graphene, are plotted in Fig. 2d^40,46^. The binding of ssDNA is evident in the top and cross-sectional views of the SEM images (refer to Fig. 2e, f). In addition, a time-of-flight secondary ion mass spectrometry (TOF-SIMS) analysis was conducted to confirm the sequencing of the sensor array (refer to Fig. 2g). Thus, the DNA sequences of thymine, adenine, and guanine exhibited nucleic acid peaks of 125 (C_5_H_5_N_2_O_2_−), 134 (C_5_H_4_N_5_−), and 150 (C_5_H_4_N_5_O−), respectively^47^. Furthermore, the proper implementation of the sensor array was confirmed through the results of a mass peak analysis according to the ssDNA mixture of the formed sensor array (Figs. S2–S7). The sequence configuration of the graphene ssDNA sensor array for sensing gases under low- and high-humidity conditions is presented in Fig. 3a; the results demonstrated that the ssDNA chemically doped the graphene through a change in the G peak of the Raman spectra of graphene after ssDNA binding; thus, the sensor array was properly implemented (refer to Fig. 3b–f).

### Sensing characteristics

The measurement conditions for the target gas under dry conditions are listed in Table S1. For all three types of individual gases, the three mixed gases were measured for three distinct concentrations: the NO_2_, NO, and NH_3_ gases were measured at 2, 5, and 10 ppm, respectively, and the mixed gas was measured by varying the mixture ratio.

The 1:1 mixing ratio was measured at 2, 5, and 10 ppm and was optimized at 10 ppm. Consequently, a concentration of 10 ppm was kept constant for mixing ratios of 2:1, 1:2, 3:1, and 1:3. The individual gases were measured three times for each concentration, and the mixed gas was measured nine times per mixing ratio. The gas measurement was performed for 368 s and had a recovery time of 114 s. Our sensors detected small concentrations of gas with a fast response and recovery time (refer to Table S2)^15,48–81^. The reactivity of the graphene-ssDNA gas sensor array for NH_3_ and NO_2_ is presented in Fig. 3g–j.

The seven-sensor array exhibited a reactivity of approximately 5–7.8% for NH_3_ gas; notably, the reaction rate increased with an increase in concentration (refer to Fig. 3g). A sensor consisting of a combination of ssDNA T6 and G6 with an NH_3_ concentration of 10 ppm exhibited a reactivity of approximately 15%. In contrast, the response of the sensor array with NO_2_ (refer to Fig. 3h) exhibited a slightly lowered response of 5–12%, whereas the array comprising the DNA sequence demonstrated the highest reactivity with a response of approximately 12% with a graphene-A6 sequence. According to the difference in reactivities, data from a pristine graphene sensor suggested a diminished performance under an in situ change in concentration and selectivity compared to that of a DNA-functionalized sensor, at approximately 10% for NH_3_ and NO_2_. Generally, the sensor array can contribute to the improvement in selectivity by varying its reactivity for each gas^82,83^. The ssDNA-functionalized sensor had an improved selectivity for these two gases via graphene functionalization. In addition to the responsiveness of the sensor array, different features of the sensor array can be applied for gas discrimination (refer to Fig. 3i, j). The time constant is a feature related to the reaction diagram, and the area is a feature related to the reaction rate and reactivity. Evidently, the extracted features from the sensor data exhibit selectivity depending on the type of gas, which improves the classification rate for the determination of a specific gas type. Thereafter, to determine the mixed gas composition, the gas mixture ratios were measured. For a mixing ratio of 1:1, the sum of the two gas concentrations was measured at 2, 5, and 10 ppm, and the reactivity gradually improved as the concentration increased. According to the data, the reactivity changed slightly according to the mixing ratio; this trend is identical to that observed for the PCA plot. However, no obvious difference in reactivity was observed. We chose to use artificial intelligence for analysis to address this issue.

The measurements of NH_3_ and H_2_S were conducted under humid conditions to determine the applicability of the sensor in breath analyzers. In an environment similar to that of exhaled breath (80% humidity), individual gas measurements at concentrations of 2, 5, and 10 ppm were repeated three times for 368 s with an air recovery time of 114 s. Mixing ratios of 1:1, 1:3, and 3:1 were measured nine times at a concentration of 10 ppm to discriminate between the gas species under humid and dry conditions. Consequently, compared to those under dry conditions, we obtained an improved reactivity for NH_3_ (20–32%) and H_2_S (20–40%) under humid conditions. As with the data under dry conditions, these data were also analyzed using artificial intelligence.

### Chemical vapor discrimination with feature selection

**Fig. 4:**
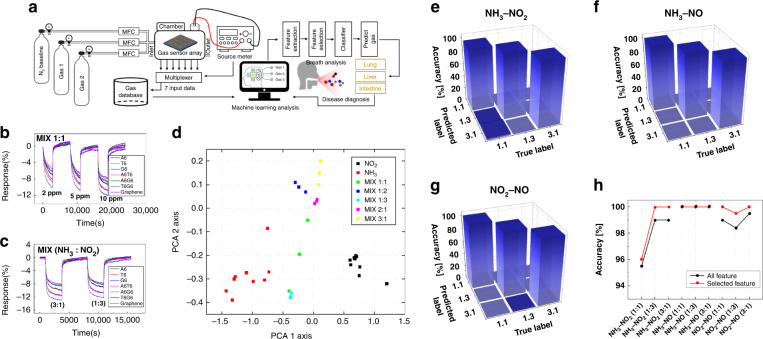
SVM classifications of low-humidity gases. Schematic diagram (**a**) for gas data acquisition and gas species identification. **b** Mixed gas at a 1:1 ratio of NH_3_ to NO_2_ and **c** data at mixture ratios of 1:3 and 3:1. **d** PCA plots after feature extraction: the data exhibit a constant degree of dispersion for the mixed gas. Confusion matrix for **e** NH_3_–NO_2_, **f** NH_3_–NO, and **g** NO_2_–NO mixed gases after feature selection. **h** Comparison of the classification rate of the Boruta algorithm with feature selection and SVM analysis without feature selection under conditions of low humidity

The incorporation of appropriate features significantly positively impacts the performance of a machine learning model^84,85^. A machine learning model possesses fewer hyperparameters than a deep learning model. Therefore, to fine-tune this hyperparameter and obtain the best performance, features with minimum dimensions should be extracted while maintaining as much of the data as possible. The features extracted from the response data of the gas sensor array should be representative, exhibit appropriate physical and chemical significance, and be able to account for the correlation between data points. In this study, we used the Boruta algorithm for feature selection (refer to Figure S15); the dimensions of the extracted features were reduced by using this technique, and it was subsequently applied for classification. The process of sensing the mixed gas and discriminating the gases with feature selection is illustrated in Fig. 4a. To analyze the effect of the feature selection technique, support vector machine (SVM) analysis was conducted by implementing the extracted features without feature selection; the results of feature selection were generally higher than those without such an application.

Some errors in the form of noise may occur during gas measurements for several reasons, and we applied the filter for correction. First, ambient white noise may be introduced into the measurements. Second, noise occurs owing to the processes pertaining to the gases in the chamber, such as desorption during the adsorption process or adsorption during the desorption process between gas measurements. Third, noise occurs owing to the time difference of the input voltage in the multichannel switching device during sensor array measurement. Fourth, thermal noise is generated by the heaters used in the metal oxide gas sensor, which is introduced into the measurements. Finally, discrete reactions occur for certain gases in certain types of sensors. Therefore, we employed the filtfilt function in the Python 3.7 package SciPy 1.4.1, which is a forward-backward filter for correcting such noise. This linear filter achieves zero-phase filtering by applying an infinite impulse response filter twice—once forward and once backward. All the hyperparameters in the filtfilt function were optimized. The results of the filter were as follows: We extracted a total of 840 features to increase the recognition rate for gas species, with 120 features for each of the seven sensors. The feature sets included the magnitude, derivative, difference, time constant, and area under the graph; these feature sets are listed in detail in Table S3. The magnitude included the maximum absolute value, which can be the maximum or minimum magnitude depending on the selectivity performance of the sensor, downsampled values of the magnitude, and downsampled values of the normalized magnitude. The derivative included the maximum and minimum derivatives of the graph, downsampled values of the derivative, and maximum and minimum second derivatives during both the injection and purging stages. The differences were calculated on the basis of five intervals (two in the reaction stage and three in the purging stage). The time constant and area were calculated for eight intervals (four in the reaction stage and four in the purging stage), and the time constant was calculated based on the start and end points of each reaction. However, in the MDFG data, certain features exhibited minimal selectivity for certain gases, and certain data were more noise-like than those of the reactions. These characteristics prevented our feature extraction algorithms from performing effectively. Therefore, we set the feature value of the low-quality data to zero; this irrelevant feature was removed during the feature-selection stage.

Feature selection is an essential technique for machine learning. By eliminating highly correlated, irrelevant, and noisy features, the occurrence of overfitting is reduced, and the performance of the model is improved by minimizing the variance and maximizing the generalizability of the model. The efficiency of this algorithm can be further improved by reducing the operation time and computational load during feature selection. We considered three types of mixed gases with various mixing ratios, namely, NO_2_–NH_3_, NO–NH_3_, and NO–NO_2_, with mixing ratios of 1:1, 1:3, and 3:1 under low-humidity conditions. The gas response data from the sensor array, which are depicted in Fig. 4b and c, were input to the model, and the model output the mixture ratio. The principal component analysis (PCA) results for NO_2_–NH_3_ mixed gas are illustrated in Fig. 4d. The results were obtained after optimization of the hyperparameters, such as the percentile using the Boruta algorithm and soft margin parameter using an SVM. According to the Boruta algorithm with feature selection, for the NO_2_–NH_3_ mixture, the average optimal number of selected features was 102.73. In addition, the compression ratio was 7.01. The classification accuracy from the Monte Carlo cross-validation (MCCV) was 98.67%. For the NO–NH_3_ mixture, the average optimal number of selected features was 155.79. Moreover, the compression ratio was 4.62, and the classification accuracy of the MCCV was 100%. Finally, for the NO–NO_2_ mixture, the average optimal number of selected features was 119.6, the compression ratio was 6.02, and the classification accuracy of the MCCV was 99.33%. Confusion matrices of the classification accuracy for each gas mixture are illustrated in Fig. 4e–g and provided in Table S4. The results of SVM analysis using the extracted features without feature selection, such as the precision, recall, and f1 score, are provided in Table S5 with the results of classification with feature selection. The classification rate increased after feature selection, and a considerably high recognition rate was obtained, as depicted in Fig. 4h.

**Fig. 5:**
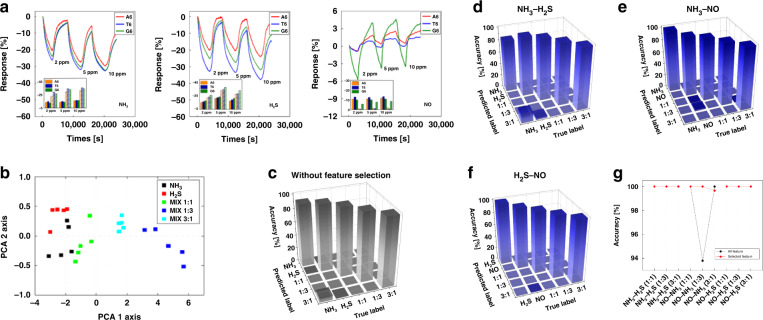
SVM classifications of high-humidity gases. **a** Reactivity for individual gas molecules of NH_3_, H_2_S, and NO at 2, 5, and 10 ppm and enhanced responses through FG-ssDNA under humid conditions. The bar with the dashes shows the reactivity in the case of 80% humidity, where an approximately 2 to 2.5 times higher reactivity is observed. Result of the subsequent **b** PCA and **c** result of SVM analysis for NH_3_-H_2_S mixed gases. The confusion matrix for **d** NH_3_-H_2_S, **e** NH_3_-NO, and **f** H_2_S-NO mixed gases after feature selection under conditions of high humidity. **g** Comparison of the classification rate of the Boruta algorithm with feature selection and SVM analysis without feature selection under conditions of high humidity

We considered five types of mixed gas, NH_3_–NO, NH_3_–H_2_S, and H_2_S–NO, with three mixed gases with mixing ratios of 1:1, 1:3, and 3:1, and two individual gases under high-humidity conditions, as depicted in Fig. 5a. The PCA and SVM analysis results for the NH_3_ and H_2_S mixed gas are illustrated in Fig. 5b and c. A comparison of the classification accuracy of the two algorithms is provided in Table S6 and Fig. 5g, and the results of feature selection under high-humidity conditions indicated higher accuracy than SVM analysis with low-humidity conditions. Therefore, we confirmed that our Boruta algorithm using feature selection for gas mixture discrimination performed better than the SVM analysis using only extracted features. According to these results, our machine learning-based feature selection algorithm can discriminate gas mixtures with high accuracy and offers high performance in high-humidity conditions, which suggests that it can be effectively applied in exhaled breath analyzers for the diagnosis of diseases.

### Chemical vapor discrimination with 1D CNN

The gas classification results of the machine learning algorithm through feature extraction and the SVM method were almost perfect; nevertheless, in this study, we designed a deep learning algorithm by employing a 1D convolutional neural network (CNN) for completely automated and rigorous mixed gas classification^86^. For the electrical signals of the experimental gas sensor array, 1D CNN models were designed for conditions of high and low humidity; these models were trained and tested for each mixed gas because the number of classes for classifying each humidity condition was different. For the low-humidity condition model, three classes of classification were performed with mixing ratios of 1:1, 1:3, and 3:1 for each mixed gas combination of NH_3_–NO, NH_3_–NO_2_, and NO_2_–NO. For the model under high-humidity conditions, five classes of classification were performed with two individual gases, and the mixing ratios for each mixed gas combination of NH_3_–NO, NH_3_–H_2_S, and H_2_S–NO were 1:1, 1:3, and 3:1.

Because the height of the data was not considered for the classification of the types and proportions of the mixed gas, the height of the data was unified to 1 through normalization, and the data were augmented with noise because the volume of experimental data for training was considerably small. The data were randomly split into a training set and test set at a ratio of 2:1 for all experimental conditions.

**Fig. 6:**
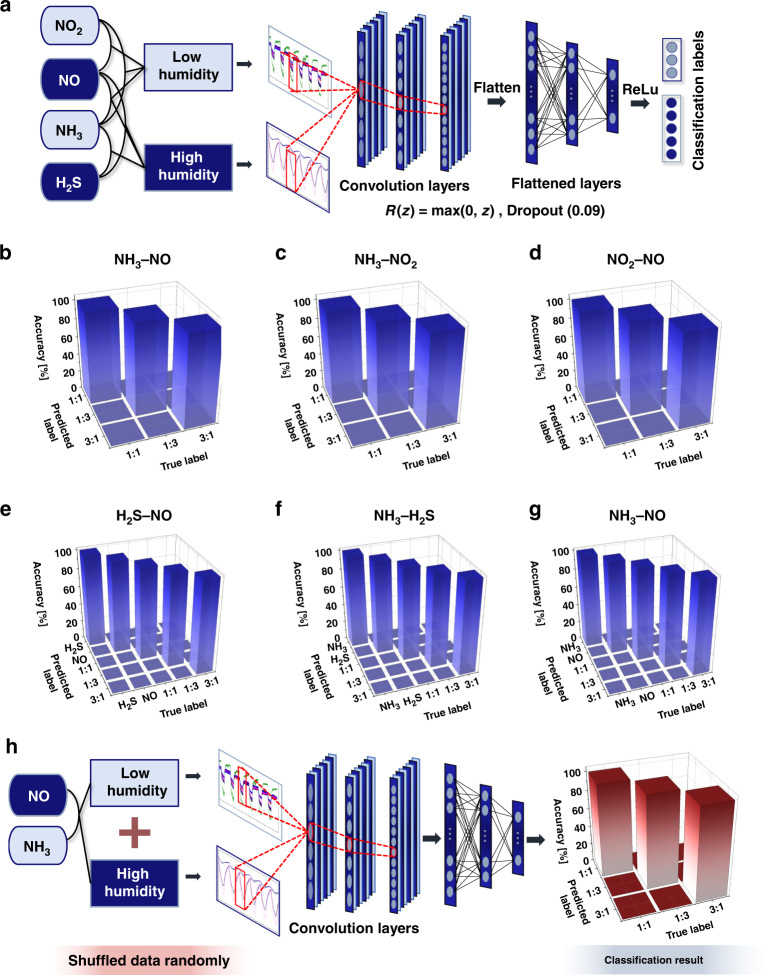
1D CNN classifications of chemical vapor compositions. **a** 1D CNN-based deep-learning structural schematic for gas classification. Confusion matrices for classification results of **b** NH_3_–NO, **c** NH_3_–NO_2_, and **d** NO_2_–NO mixed gas under low humidity conditions and at mixing ratios of 1:1, 1:3, and 3:1. Results of **e** H_2_S–NO, **f** NH_3_–H_2_S, and **g** NH_3_–NO mixed gas under conditions of high humidity and individual gas at mixing ratios of 1:1, 1:3, and 3:1. **h** NH_3_-NO mixed gas 1D CNN classification result for data randomly shuffled with humidity conditions

A schematic of the common structure of the models for low- and high-humidity conditions is presented in Fig. 6a. In the high- and low-humidity models, the processed data were input into a 1D convolution layer with a sensor array as the channels, and after three sets of convolution, activation, and dropout, the data were normalized. Then, the normalized data were input into a linear layer sequence, with three sets of linear, activation, and dropout^41,86,87^. The designed 1D CNN model was trained by repeating 20 epochs by inputting the training set, and the model was optimized through logit and loss functions. As the training loss converged to 0 for a learning rate of 0.001, the results confirmed that our model was trained well with the experimental data. We checked our model performance by inputting the test set into the trained model, and the test loss converged to 0, which was similar to that observed for the training loss.

The evaluation results of our 1D CNN model on the test set are presented as confusion matrices and illustrated in Fig. 6b–d under low-humidity conditions and in Fig. 6e–g under high-humidity conditions. Although the training and test sets were randomly divided such that they did not overlap during dataset classification, a loss of 0% and a classification accuracy of 100% were achieved. This result was observed because the data possessed different characteristics depending on the chemical vapor type and mixing ratio of the chemical vapor composition.

The classification accuracy results for all humidity conditions and mixed gases using the 1D CNN method were 100%, and these classification accuracy results are provided in Tables S4 and S6 for comparison with the machine learning-based feature selection analysis results. Therefore, we confirmed that the 1D CNN algorithm performed much better, with more stable and perfect classification accuracy. According to the results of this evaluation, mixed gas can be classified perfectly without arbitrary feature selection by using the 1D CNN model developed in this study. Therefore, we achieved the development of a deep learning algorithm for mixed gas classification that is more automated and offers greater accuracy compared to machine learning. This algorithm is expected to be the basis for the development of an automatic disease diagnosis system using human exhaled breath in the future.

Furthermore, we additionally verified the discriminative performance of our 1D CNN deep learning on mixed gas-sensing data randomly mixed for low- and high-humidity conditions. Data sensed by our MDFG sensor were used under low and high-humidity conditions for NH_3_–NO gas mixed at 1:1, 1:3, and 3:1 for deep learning performance verification. Data were randomly shuffled, and the combination of NH_3_–NO mixed gas was discriminated. As a result, our deep learning achieved 100% test results from the model. We set most conditions of the model to be the same as those of our previous model to target the performance verification of our deep learning model, and the deep learning analysis process and classification results are shown in Fig. 6h. Thus, our 1D CNN model has been shown to be able to classify a combination of mixed gases even under random humidity conditions. As the humidity contained in human exhaled breath and natural surroundings is not constant, this result is very important. Since the aim of this study is to introduce a new device and approach for future investigation and commercial purposes, this result may suggest that this study is one step closer to achieving that objective.

## Discussion

We initially present the results of mixtures of gas distinguished with an early ratio under the real conditions of human exhaled breath with AI integrated with an arrayed-sensor platform. The characterization and analyses of gas mixtures under humid conditions are critical to the application of sensors in the diagnosis of diseases through the analysis of exhaled breath. Although typical sensors may not be capable of detecting chemical vapor composition under humid conditions, our graphene-based DNA-functionalized gas sensors exhibited high reactivity even in humid environments. We achieved a reactivity of up to 20% under humid conditions through the application of a gas sensor array via the ssDNA-functionalization of graphene. For the accurate discrimination of chemical vapor composition, we proposed two powerful artificial intelligence techniques. Feature selection analysis achieved more than 98% classification accuracy under humid conditions, but the 1D CNN model showed perfect classification results. Therefore, we found that our 1D CNN model was capable of perfect mixed gas classification without arbitrary feature selection. The results obtained in this study demonstrated that a sensor array and artificial intelligence-aided discrimination techniques can be applied synergistically in a real-time gas discrimination system and for the early diagnosis of diseases through exhaled breath. Furthermore, this study provides a new approach for the detection or verification of gas molecule species in other applications.

## Materials and methods

### Sample preparation and characterization

A negative photoresist (AZ-5214™) was patterned using photolithography to manufacture the electrode contact. Metal sputter deposition (Cr: 10 nm; Au: 50 nm) was performed, and a lift-off process was utilized to deposit the contact metal. The sensing component was fabricated via graphene transfer. Graphene was grown at 1000 °C under methane and hydrogen gas pressures of 285 and 40 mTorr, respectively, for 10 min. After growth, the graphene was left undisturbed to cool to room temperature. After coating poly(methyl methacrylate) (PMMA) through a spin-coating method, monolayer graphene was grown on Cu foil at 4200 rpm and baked at 180 °C for 2 min. The copper was subsequently removed using a Cu etchant (ammonium persulfate, Sigma-Aldrich, A3678) and transferred to a Si wafer in deionized water; the PMMA was thereafter removed using acetone. Single-stranded DNA (Xenotech Co.) was used to functionalize the graphene by utilizing a droplet under conditions of 100% relative humidity for 3 h. The thickness of the device was determined via AFM (Atomic Force Microscope, Park Systems, NX-10), and TOF-SIMS (Secondary Ion Mass Spectrometry) was implemented to characterize the ssDNA (TOF SIMS 5; IONTOF).

### Sensor array measurements

The gas measurements were conducted in a chamber with electrode tips. A constant voltage (0.1 V) was applied to the electrode tips, and the change in conductance was monitored by using a source measurement unit (Keithley 2602). For the measurements, the gas flow was maintained at 1000 sccm, and the gases were controlled through mass-flow controllers. The baseline gas was N_2_ (99.9%), which was employed as the baseline gas, and 50 ppm N_2_ was utilized as the target gas.

### Boruta algorithm

To classify the gas types with feature selection, we used the Boruta algorithm. Boruta is a wrapper algorithm that is used in random forests. This algorithm is implemented via the following procedures: copies of all features are added to the dataset (which are called shadow features), they are shuffled, the random forest classifier is trained for an extended dataset, and the feature importance scores are finally gathered, which represent the Z scores. The importance of the real features is verified by comparing the Z scores of the real features against the maximum Z score of the shadow feature and removing those real features with Z scores lower than their shadow features. This process is repeated until importance is assigned to all features or until the algorithm reaches a specific set limit for the random forest runs. Based on the Boruta algorithm, the features were divided into confirmed and rejected features.

### Support vector machine analysis

To classify the gas types without feature selection, we implemented an SVM. The working principle of an SVM is to find a hyperplane that separates the features to the maximum extent using nonlinear mapping. Accordingly, the SVM considers features from different classes as points in the high-dimensional feature space. Although SVMs were originally designed for binary classification, they can be extended to multiclass classification by combining multiple binary SVMs. In particular, SVM guarantees the global optimality of the trained model, which is a major advantage. To assess the generality of the trained model, we used a cross-validation technique. Owing to the small size of the dataset, the Monte Carlo cross-validation (MCCV) technique was more feasible than the k-fold cross-validation technique. MCCV is a validation technique wherein random subsampling validation is iterated. In the MCCV technique, the gas dataset was split into training and test sets, the model was fitted to the training data and evaluated through the test data, and the average accuracy was obtained by repeating the procedure for a sufficient number of iterations.

Thus, the data were used efficiently, and the models were evaluated for approximately all possible use cases. The greater the number of iterations applied, the greater the number of cases that can be considered. In addition, we compared the accuracies of the two types of models for gas species analysis—an SVM model using feature selection techniques and another with raw data features.

### 1D convolutional neural network analysis

In this study, we used a 1D CNN, which is a deep learning technique used to extract features through convolution during the data scanning process for one-dimensional vector-type data to classify the gas species in a mixture of chemical vapors. This technique offers high efficiencies because it reduces the scan range to data approximate to the feature through iterative convolution in the presence of a repeating feature that is similar to that of the experimental data^41^. We utilized the Python 3.7 and PyTorch 1.10.0 framework to create a 1D CNN architecture.

For the experimental data, true classes were generated using a one-hot encoding method for mixed-gas classification. We set a kernel size of 7, stride size of 1, training batch size of 64, test batch size of 16, learning rate of 0.0004, dropout of 0.09, and first input channels of the 1D convolution layer of our sensor array for the low- and high-humidity condition models. We employed the rectified linear unit function as the activation function, the binary cross-entropy function as the loss function, and the Adam optimizer as the optimizer for the PyTorch framework.

Because the model was applied as a test set, the weight model featuring the minimum loss was applied in the PyTorch 1.10.0 framework for model evaluation. The loss was converted to the probability for one-hot encoding classes by using the sigmoid function, and the class corresponding to the position with a probability of 0.5 or more was used as the prediction class.

## Supplementary information

Supplementary Information

## References

[1] Liu X (2012). A survey on gas sensing technology. Sensors.

[2] Bhushan, B. *Springer Handbook of Nanotechnology* (Springer, 2017).

[3] Huang C, Notten A, Rasters N (2011). Nanoscience and technology publications and patents: a review of social science studies and search strategies. J. Technol. Transf..

[4] Sattler, K. D. *Handbook of Nanophysics: Principles and Methods* (CRC Press, 2010).

[5] Lourenço C, Turner C (2014). Breath analysis in disease diagnosis: methodological considerations and applications. Metabolites.

[6] Morisco F (2013). Rapid “breath-print” of liver cirrhosis by proton transfer reaction time-of-flight mass spectrometry. A pilot study. PLoS ONE.

[7] Choi J, Hoffman LA, Rodway GW, Sethi JM (2006). Markers of lung disease in exhaled breath: nitric oxide. Biol. Res. Nurs..

[8] Li L, Moore P (2008). Putative biological roles of hydrogen sulfide in health and disease: a breath of not so fresh air?. Trends Pharmacol. Sci..

[9] Morselli-Labate AM, Fantini L, Pezzilli R (2007). Hydrogen sulfide, nitric oxide and a molecular mass 66 u substance in the exhaled breath of chronic pancreatitis patients. Pancreatology.

[10] Van den Velde S, Nevens F, van Steenberghe D, Quirynen M (2008). GC–MS analysis of breath odor compounds in liver patients. J. Chromatogr. B.

[11] Wang P, Zhang G, Wondimu T, Ross B, Wang R (2011). Hydrogen sulfide and asthma. Exp. Physiol..

[12] Güntner AT, Righettoni M, Pratsinis SE (2016). Selective sensing of NH_3_ by Si-doped α-MoO_3_ for breath analysis. Sens. Actuators B: Chem..

[13] Lone MY (2019). Fabrication of sensitive SWCNT sensor for trace level detection of reducing and oxidizing gases (NH_3_ and NO_2_) at room temperature. Phys. E: Low. Dimens. Syst. Nanostruct..

[14] Zhang L (2019). Highly sensitive NH_3_ wireless sensor based on Ag-RGO composite operated at room-temperature. Sci. Rep..

[15] Yang L (2020). Novel gas sensing platform based on a stretchable laser-induced graphene pattern with self-heating capabilities. J. Mater. Chem. A.

[16] Yi N, Shen M, Erdely D, Cheng H (2020). Stretchable gas sensors for detecting biomarkers from humans and exposed environments. TrAC Trends Anal. Chem..

[17] Ko G-J (2020). Biodegradable, flexible silicon nanomembrane-based NO_x_ gas sensor system with record-high performance for transient environmental monitors and medical implants. NPG Asia Mater..

[18] Molina A (2022). Efficient NO_2_ detection and the sensing mechanism of stretchable/biodegradable MWCNT based sensors decorated with CeO_2_ nanoparticles. Synth. Met..

[19] Capone S (2003). Solid state gas sensors: state of the art and future activities. J. Optoelectron. Adv. Mater..

[20] Sung, S.-H. & Jun, S. C. *Electronic Nose System Technology for Gas Molecule Identification and Analysis by Time Series Eigengraph Analysis Based on Artificial intelligence*. Master thesis, Yonsei University (2021).

[21] Yang L (2022). Moisture-resistant, stretchable NO_x_ gas sensors based on laser-induced graphene for environmental monitoring and breath analysis. Microsyst. Nanoeng..

[22] Gupta Chatterjee S, Chatterjee S, Ray AK, Chakraborty AK (2015). Graphene–metal oxide nanohybrids for toxic gas sensor: a review. Sens. Actuators B: Chem..

[23] Meng F-L, Guo Z, Huang X-J (2015). Graphene-based hybrids for chemiresistive gas sensors. TrAC Trends Anal. Chem..

[24] Toda K, Furue R, Hayami S (2015). Recent progress in applications of graphene oxide for gas sensing: a review. Anal. Chim. Acta.

[25] Varghese SS, Lonkar S, Singh KK, Swaminathan S, Abdala A (2015). Recent advances in graphene based gas sensors. Sens. Actuators B: Chem..

[26] Geim, A. K. & Novoselov, K. S. in *Nanoscience and Technology: A Collection of Reviews from Nature Journals* 11–19 (World Scientific, 2010).

[27] Basu S, Bhattacharyya P (2012). Recent developments on graphene and graphene oxide based solid state gas sensors. Sens. Actuators B: Chem..

[28] He Q, Wu S, Yin Z, Zhang H (2012). Graphene-based electronic sensors. Chem. Sci..

[29] Liu Y, Dong X, Chen P (2012). Biological and chemical sensors based on graphene materials. Chem. Soc. Rev..

[30] Yavari F, Koratkar N (2012). Graphene-based chemical sensors. J. Phys. Chem. Lett..

[31] Yuan W, Shi G (2013). Graphene-based gas sensors. J. Mater. Chem. A.

[32] Choi YR (2015). Role of oxygen functional groups in graphene oxide for reversible room-temperature NO_2_ sensing. Carbon.

[33] Mattson E (2015). Investigation of NO_2_ adsorption on reduced graphene oxide. Chem. Phys. Lett..

[34] Chung MG (2012). Highly sensitive NO_2_ gas sensor based on ozone treated graphene. Sens. Actuators B: Chem..

[35] Hong J (2015). A highly sensitive hydrogen sensor with gas selectivity using a PMMA membrane-coated Pd nanoparticle/single-layer graphene hybrid. ACS Appl. Mater. Interfaces.

[36] Niu F, Tao L-M, Deng Y-C, Wang Q-H, Song W-G (2014). Phosphorus doped graphene nanosheets for room temperature NH_3_ sensing. N. J. Chem..

[37] Pak Y (2014). Palladium-decorated hydrogen-gas sensors using periodically aligned graphene nanoribbons. ACS Appl. Mater. Interfaces.

[38] Koziej D (2005). Water–oxygen interplay on tin dioxide surface: implication on gas sensing. Chem. Phys. Lett..

[39] Vlachos D, Skafidas P, Avaritsiotis J (1995). The effect of humidity on tin-oxide thick-film gas sensors in the presence of reducing and combustible gases. Sens. Actuators B: Chem..

[40] Jung Y (2017). Humidity‐tolerant single‐stranded DNA‐functionalized graphene probe for medical applications of exhaled breath analysis. Adv. Funct. Mater..

[41] Kiranyaz S (2021). 1D convolutional neural networks and applications: a survey. Mech. Syst. signal Process..

[42] cadnav. *Female Head Base Mesh 3D Model*. http://www.cadnav.com (2012).

[43] cadnav. *Location of the Human Cerebrum 3D Model*. http://www.cadnav.com (2014).

[44] Umadevi D, Panigrahi S, Sastry GN (2014). Noncovalent interaction of carbon nanostructures. Acc. Chem. Res..

[45] Georgakilas V (2012). Functionalization of graphene: covalent and non-covalent approaches, derivatives and applications. Chem. Rev..

[46] Wang QH (2012). Understanding and controlling the substrate effect on graphene electron-transfer chemistry via reactivity imprint lithography. Nat. Chem..

[47] Lee C-Y, Harbers GM, Grainger DW, Gamble LJ, Castner DG (2007). Fluorescence, XPS, and TOF-SIMS surface chemical state image analysis of DNA microarrays. J. Am. Chem. Soc..

[48] Javadian-Saraf A, Hosseini E, Wiltshire BD, Zarifi MH, Arjmand M (2021). Graphene oxide/polyaniline-based microwave split-ring resonator: a versatile platform towards ammonia sensing. J. Hazard. Mater..

[49] Liu A (2021). The gas sensor utilizing polyaniline/MoS_2_ nanosheets/SnO_2_ nanotubes for the room temperature detection of ammonia. Sens. Actuators B: Chem..

[50] Wu Q (2021). An enhanced flexible room temperature ammonia gas sensor based on GP-PANI/PVDF multi-hierarchical nanocomposite film. Sens. Actuators B: Chem..

[51] Ma J (2021). Multi-walled carbon nanotubes/polyaniline on the ethylenediamine modified polyethylene terephthalate fibers for a flexible room temperature ammonia gas sensor with high responses. Sens. Actuators B: Chem..

[52] Hien HT (2021). High NH_3_ sensing performance of NiO/PPy hybrid nanostructures. Sens. Actuators B: Chem..

[53] Wang S (2021). Ultrathin Nb_2_CTx nanosheets-supported polyaniline nanocomposite: enabling ultrasensitive NH_3_ detection. Sens. Actuators B: Chem..

[54] Hu Q (2021). Design and preparation of hollow NiO sphere-polyaniline composite for NH_3_ gas sensing at room temperature. Sens. Actuators B: Chem..

[55] Shoeb M, Mobin M, Ahmad S, Naqvi AH (2021). Facile synthesis of polypyrrole coated graphene Gr/Ag–Ag_2_O/PPy nanocomposites for a rapid and selective response towards ammonia sensing at room temperature. J. Sci.: Adv. Mater. Devices.

[56] Shahmoradi A, Hosseini A, Akbarinejad A, Alizadeh N (2021). Noninvasive detection of ammonia in the breath of hemodialysis patients using a highly sensitive ammonia sensor based on a polypyrrole/sulfonated graphene nanocomposite. Anal. Chem..

[57] Luo G, Xie L, He M, Jaisutti R, Zhu Z (2021). Flexible fabric gas sensors based on reduced graphene-polyaniline nanocomposite for highly sensitive NH_3_ detection at room temperature. Nanotechnology.

[58] Oh W-C (2021). Chemo-electrical gas sensors based on LaNiMoSe_2_ in graphene and conducting polymer PANI composite semiconductor nanocomposite. J. Electron. Mater..

[59] Feng Q, Zhang H, Shi Y, Yu X, Lan G (2021). Preparation and gas sensing properties of PANI/SnO_2_ hybrid material. Polymers.

[60] Albaris H, Karuppasamy G (2020). Investigation of NH_3_ gas sensing behavior of intercalated PPy–GO–WO_3_ hybrid nanocomposite at room temperature. Mater. Sci. Eng.: B.

[61] Amarnath M, Heiner A, Gurunathan K (2020). Size controlled V_2_O_5_-WO_3_ nano-islands coated polypyrrole matrix: a unique nanocomposite for effective room temperature ammonia detection. Sens. Actuators A: Phys..

[62] Wu T, Lv D, Shen W, Song W, Tan R (2020). Trace-level ammonia detection at room temperature based on porous flexible polyaniline/polyvinylidene fluoride sensing film with carbon nanotube additives. Sens. Actuators B: Chem..

[63] Zhang J, Wu C, Li T, Xie C, Zeng D (2020). Highly sensitive and ultralow detection limit of room-temperature NO_2_ sensors using in-situ growth of PPy on mesoporous NiO nanosheets. Org. Electron..

[64] Abun A, Huang B-R, Saravanan A, Kathiravan D, Hong P-D (2020). Effect of PMMA on the surface of exfoliated MoS_2_ nanosheets and their highly enhanced ammonia gas sensing properties at room temperature. J. Alloy. Compd..

[65] Husain A, Ahmad S, Mohammad F (2020). Electrical conductivity and ammonia sensing studies on polythiophene/MWCNTs nanocomposites. Materialia.

[66] Tanguy NR, Wiltshire B, Arjmand M, Zarifi MH, Yan N (2020). Highly sensitive and contactless ammonia detection based on nanocomposites of phosphate-functionalized reduced graphene oxide/polyaniline immobilized on microstrip resonators. ACS Appl. Mater. Interfaces.

[67] Deshmukh K, Pasha SK (2020). Room temperature ammonia sensing based on graphene oxide integrated flexible polyvinylidenefluoride/cerium oxide nanocomposite films. Polym.-Plast. Technol. Mater..

[68] Singh P, Kushwaha CS, Singh VK, Dubey G, Shukla SK (2021). Chemiresistive sensing of volatile ammonia over zinc oxide encapsulated polypyrrole based nanocomposite. Sens. Actuators B: Chem..

[69] Fan G (2020). Enhanced room-temperature ammonia-sensing properties of polyaniline-modified WO_3_ nanoplates derived via ultrasonic spray process. Sens. Actuators B: Chem..

[70] Gaikwad G, Patil P, Patil D, Naik J (2017). Synthesis and evaluation of gas sensing properties of PANI based graphene oxide nanocomposites. Mater. Sci. Eng.: B.

[71] Belkhamssa N, Ksibi M, Shih A, Izquierdo R (2020). Fabrication of fast responsive and insensitive-humidity sensor based on polyaniline-WO_3_-CuCl_2_ for hydrogen sulfide detection. IEEE Sens. J..

[72] Sahu PK, Pandey RK, Dwivedi R, Mishra V, Prakash R (2020). Polymer/Graphene oxide nanocomposite thin film for NO_2_ sensor: an in situ investigation of electronic, morphological, structural, and spectroscopic properties. Sci. Rep..

[73] Sakhare, R., Navale, Y., Jadhav, Y., Mulik, R. & Patil, V. in *Techno-Societal 2020* 1021–1029 (Springer, 2021).

[74] Wang C (2020). One-step synthesis of polypyrrole/Fe_2_O_3_ nanocomposite and the enhanced response of NO2 at low temperature. J. Colloid interface Sci..

[75] Karmakar N (2017). Room temperature NO_2_ gas sensing properties of p-toluenesulfonic acid doped silver-polypyrrole nanocomposite. Sens. Actuators B: Chem..

[76] Dhall S, Kumar M, Bhatnagar M, Mehta B (2018). Dual gas sensing properties of graphene-Pd/SnO_2_ composites for H_2_ and ethanol: role of nanoparticles-graphene interface. Int. J. Hydrog. Energy.

[77] Xiang C (2015). Ammonia sensor based on polypyrrole–graphene nanocomposite decorated with titania nanoparticles. Ceram. Int..

[78] Zhang D, Wu Z, Zong X (2019). Metal-organic frameworks-derived zinc oxide nanopolyhedra/S, N: graphene quantum dots/polyaniline ternary nanohybrid for high-performance acetone sensing. Sens. Actuators B: Chem..

[79] Chen Z-W, Hong Y-Y, Lin Z-D, Liu L-M, Zhang X-W (2017). Enhanced formaldehyde gas sensing properties of ZnO nanosheets modified with graphene. Electron. Mater. Lett..

[80] Zhao C (2018). Facile synthesis of SnO_2_ hierarchical porous nanosheets from graphene oxide sacrificial scaffolds for high-performance gas sensors. Sens. Actuators B: Chem..

[81] Liu X, Sun J, Zhang X (2015). Novel 3D graphene aerogel–ZnO composites as efficient detection for NO_2_ at room temperature. Sens. Actuators B: Chem..

[82] Star A, Joshi V, Skarupo S, Thomas D, Gabriel J-CP (2006). Gas sensor array based on metal-decorated carbon nanotubes. J. Phys. Chem. B.

[83] Tomchenko AA, Harmer GP, Marquis BT, Allen JW (2003). Semiconducting metal oxide sensor array for the selective detection of combustion gases. Sens. Actuators B: Chem..

[84] Choi, S.-I., Eom, T. & Jeong, G.-M. Gas classification using combined features based on a discriminant analysis for an electronic nose. *J. Sens.* **2016** (2016).

[85] Yan K, Zhang D (2015). Feature selection and analysis on correlated gas sensor data with recursive feature elimination. Sens. Actuators B: Chem..

[86] Zhan C, He J, Pan M, Luo D (2021). Component analysis of gas mixture based on one-dimensional convolutional neural network. Sensors.

[87] Abdoli S, Cardinal P, Koerich AL (2019). End-to-end environmental sound classification using a 1D convolutional neural network. Expert Syst. Appl..

