# Supplementary material for: Multiplexed DNA-functionalized graphene sensor with artificial intelligence-based discrimination performance for analyzing chemical vapor compositions
Source: Microsyst Nanoeng. 2023 Mar 20;9:28. doi: 10.1038/s41378-023-00499-y (PMC10025282; doi:10.1038/s41378-023-00499-y)
Supplement: Supplementary file 1 — Supplementary Information [file 41378_2023_499_MOESM1_ESM.docx]

**Supplementary Information**

**Multiplexed DNA-functionalized graphene sensor with artificial intelligence-based discrimination performance for analyzing chemical vapor compositions**

Yun Ji Hwang‡, Heejin Yu‡, Gilho Lee‡, Iman Shackery, Jin Seong, Youngmo Jung, Seung-Hyun Sung, Jongeun Choi*, and Seong Chan Jun*

Y. J. Hwang, H. Yu, G. Lee, I. Shackery, J. Seong, Y. Jung, S. H. Sung, J. Choi, S. C. Jun

School of Mechanical Engineering, Yonsei University, 50, Yonsei-ro, Seodaemun-gu, Seoul 03722, Republic of Korea

‡ These authors contributed equally.

*Corresponding author

Seong Chan Jun

Tel: +82-2-2123-5817; E-mail: [scj@yonsei.ac.kr](mailto:scj@yonsei.ac.kr)

Jongeun Choi

Tel: +82-2-2123-2813; E-mail: jongeunchoi@yonsei.ac.kr

**
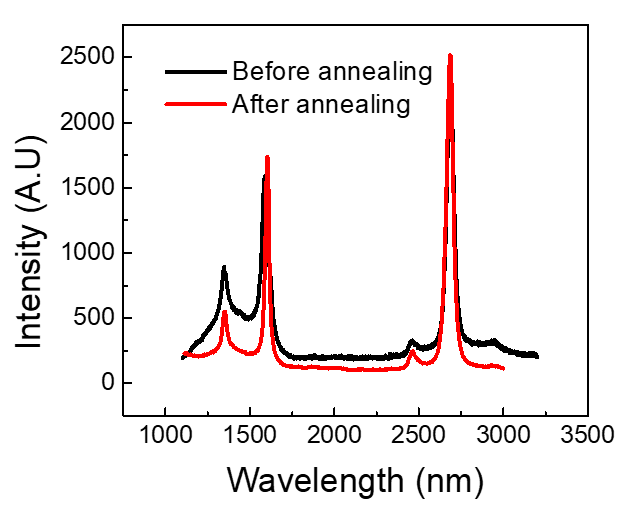
**

**Figure S1.** Changes in Raman spectra of monolayer graphene before and after annealing.


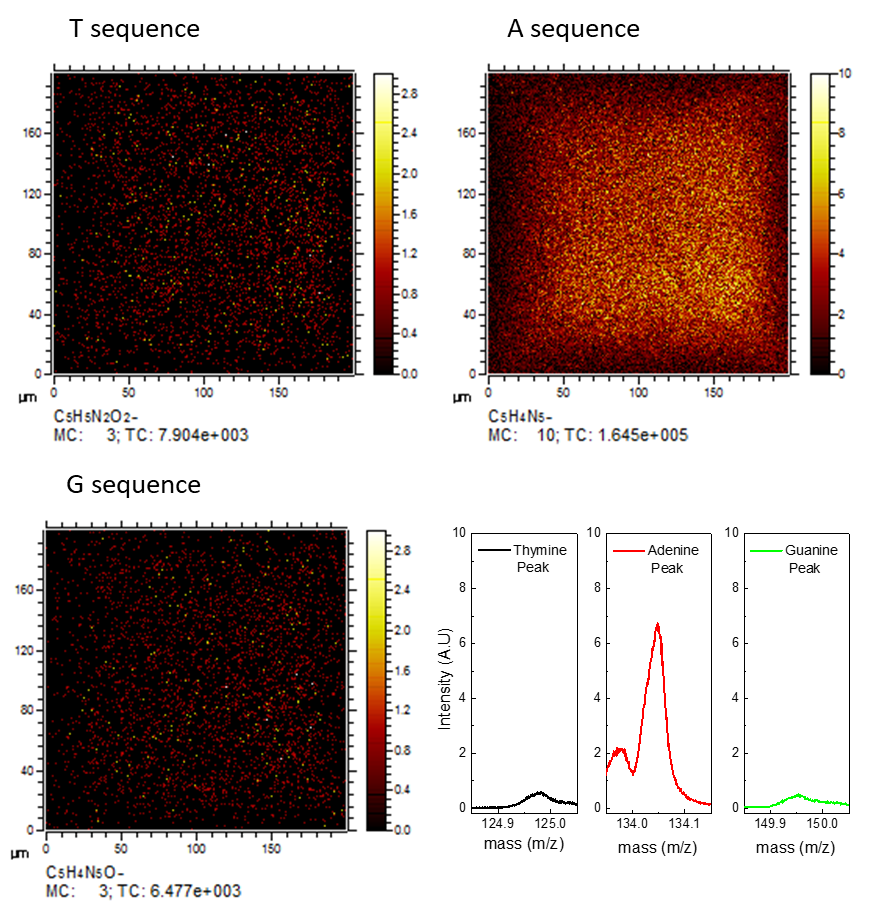


**Figure S2.** TOF-SIMS image and peak of sensing channel in A6 DNA sequence.


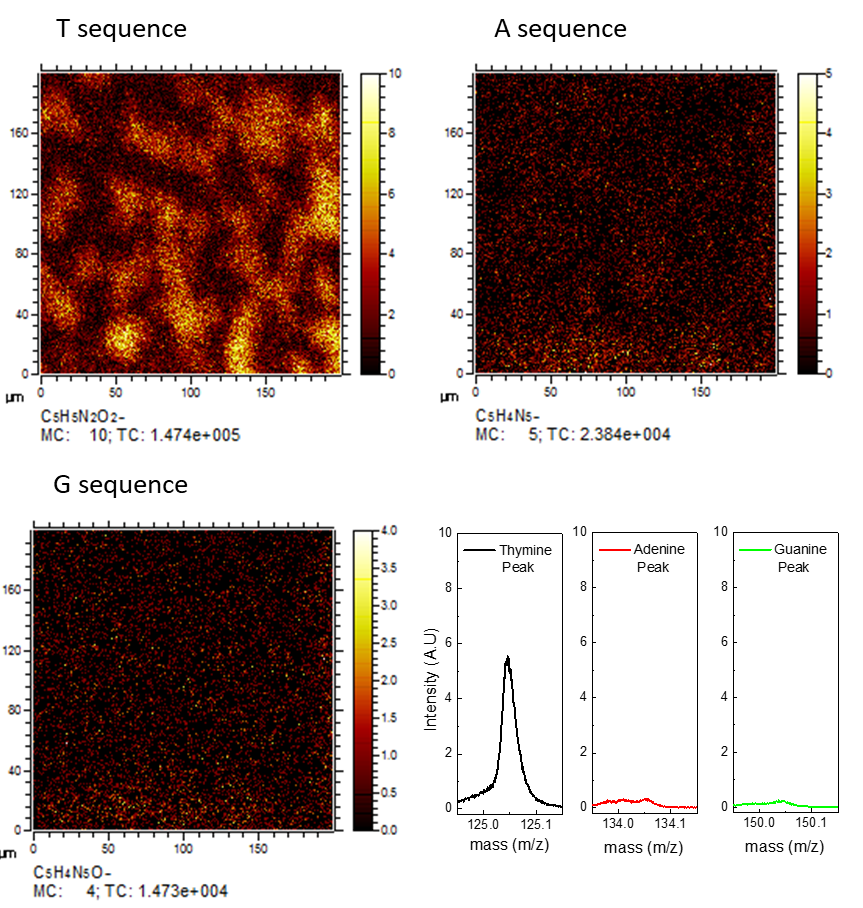


**Figure S3.** TOF-SIMS image and peak of sensing channel in T6 DNA sequence.


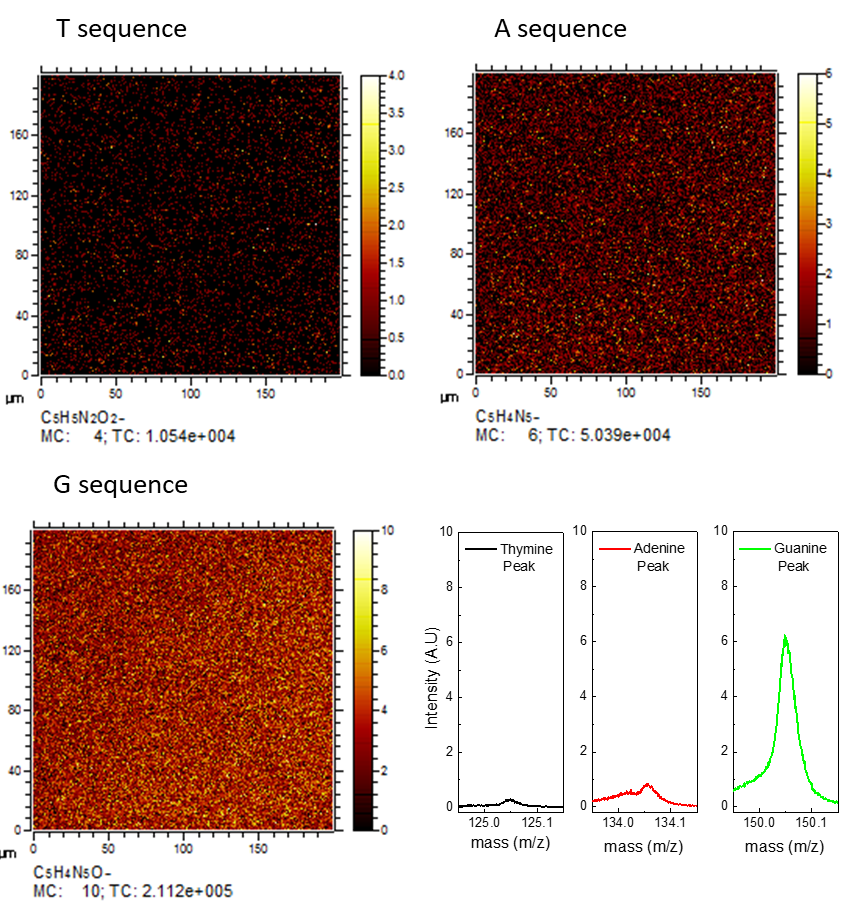


**Figure S4.** TOF-SIMS image and peak of sensing channel in G6 DNA sequence.


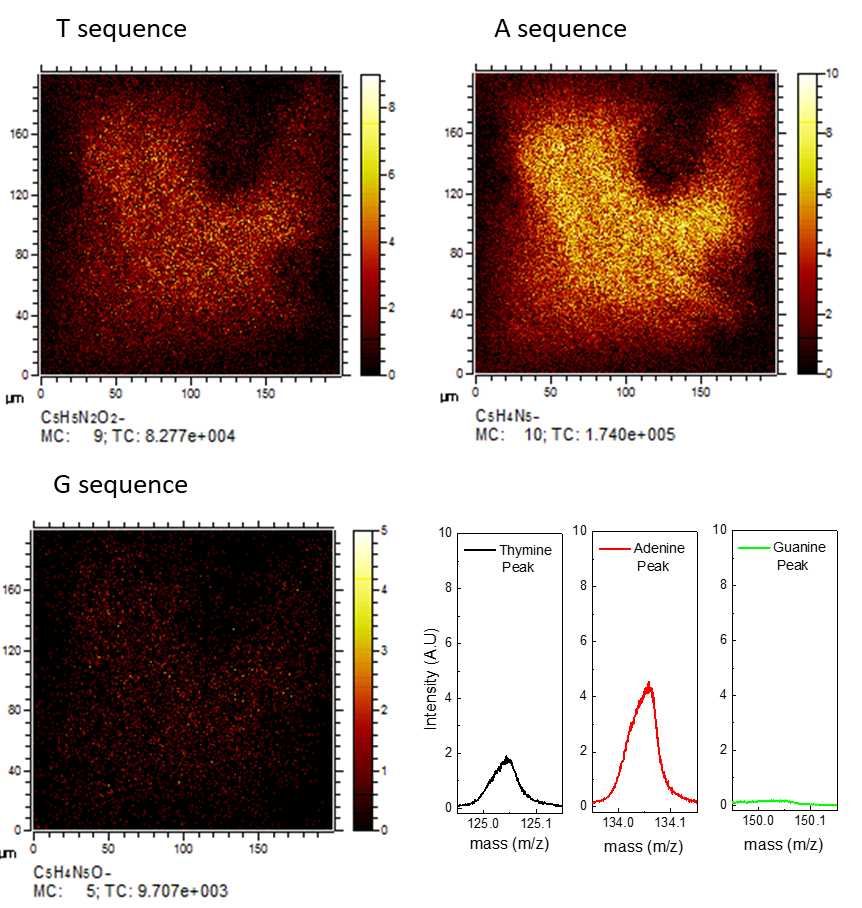


**Figure S5.** TOF-SIMS image and peak of sensing channel in A6T6 DNA sequence.


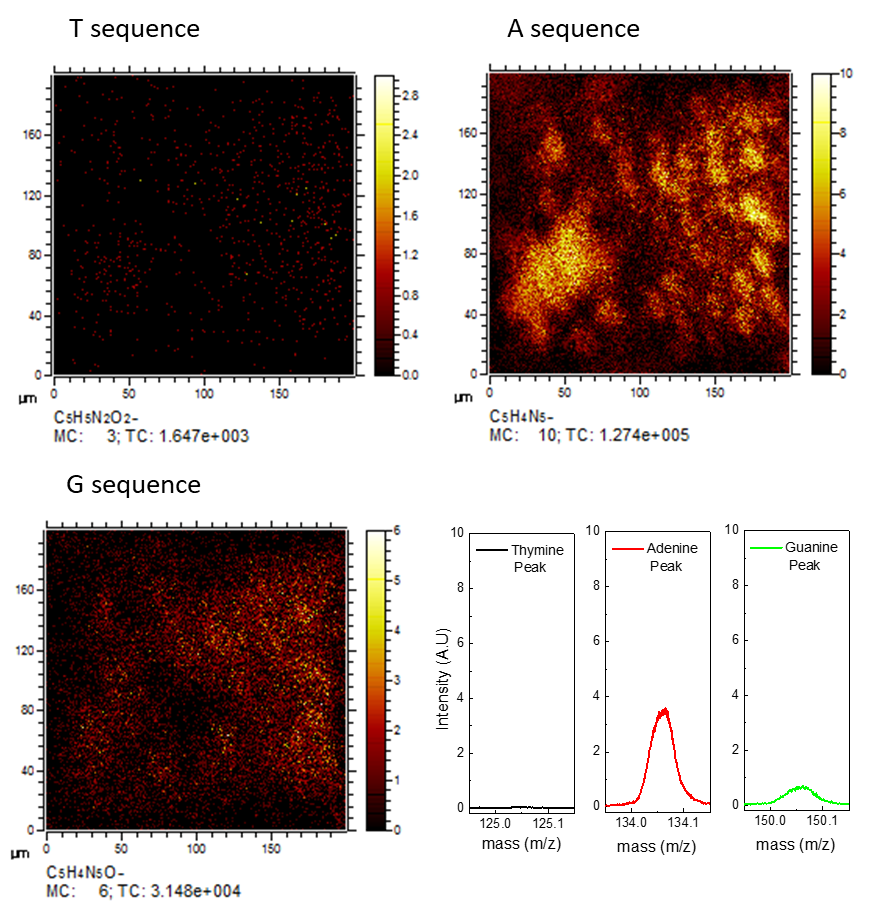


**Figure S6.** TOF-SIMS image and peak of sensing channel in A6G6 DNA sequence.


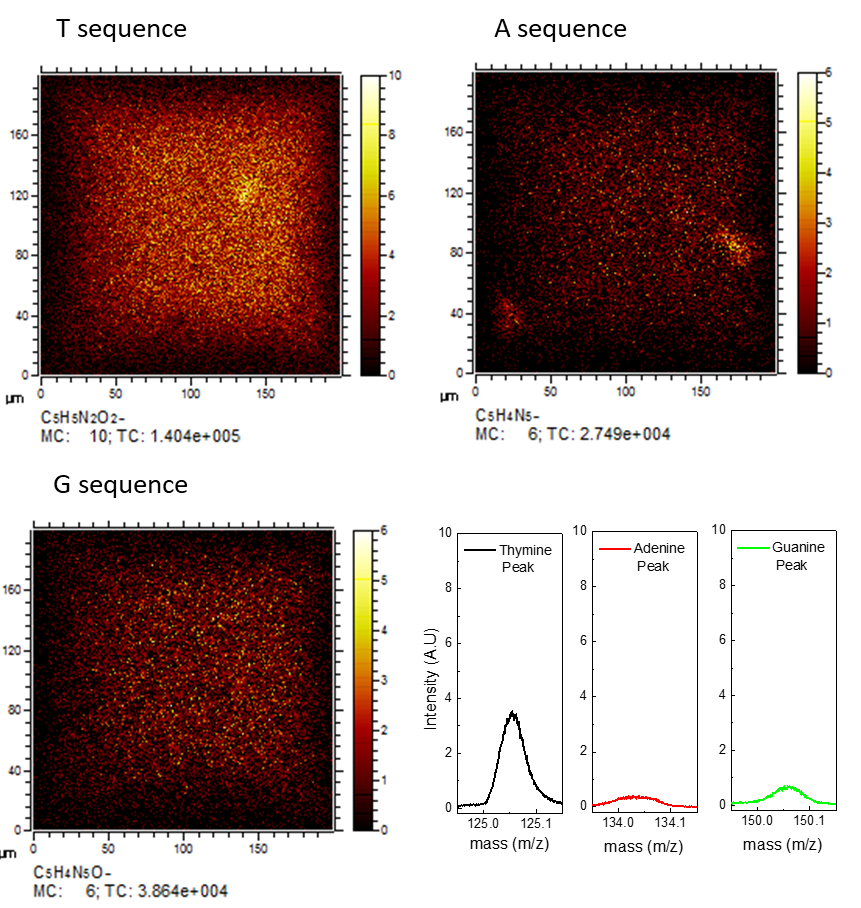


**Figure S7.** TOF-SIMS image and peak of sensing channel in T6G6 DNA sequence.


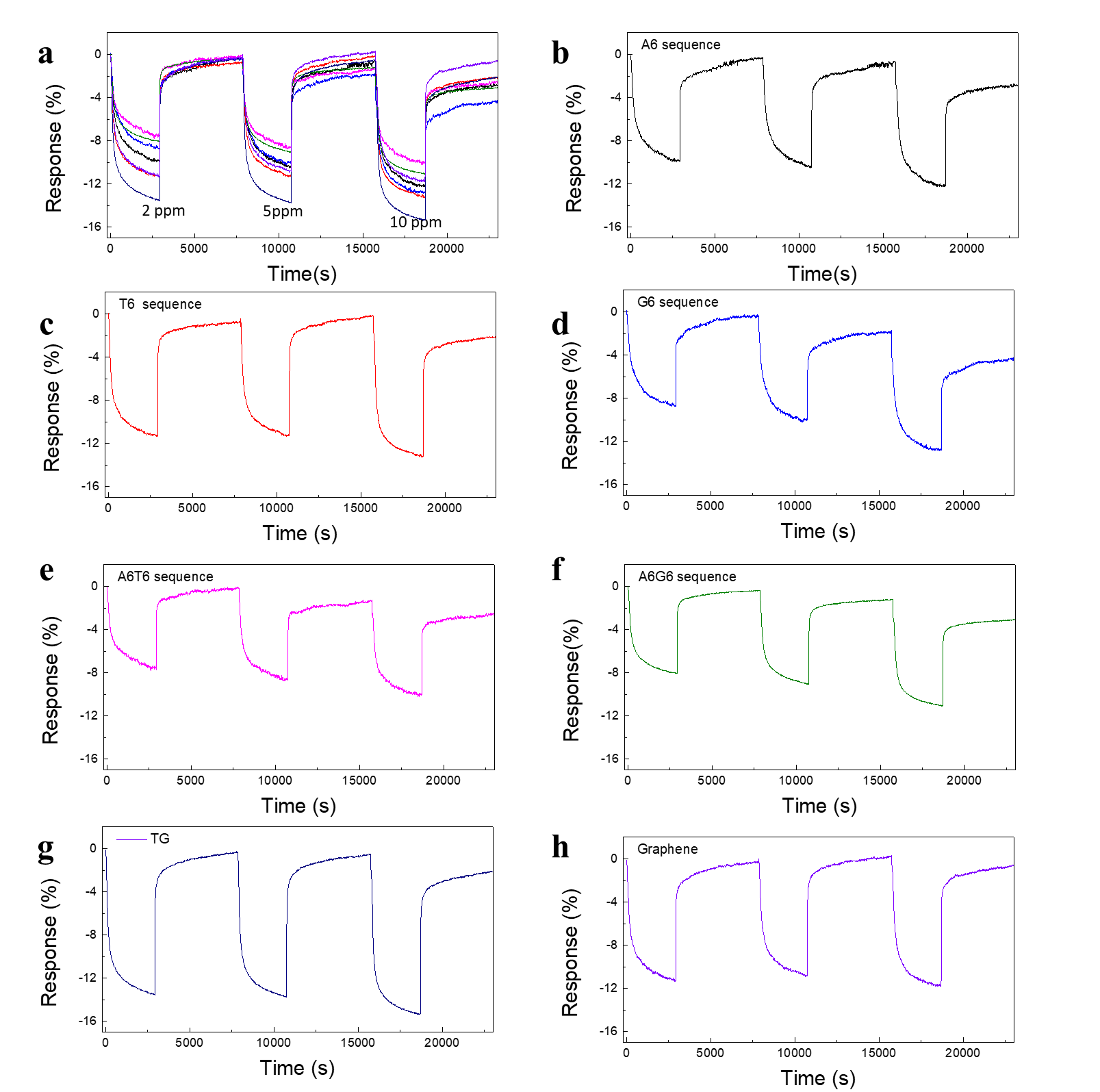


**Figure S8.** Response of NH_3_ individual gas under low-humidity condition.


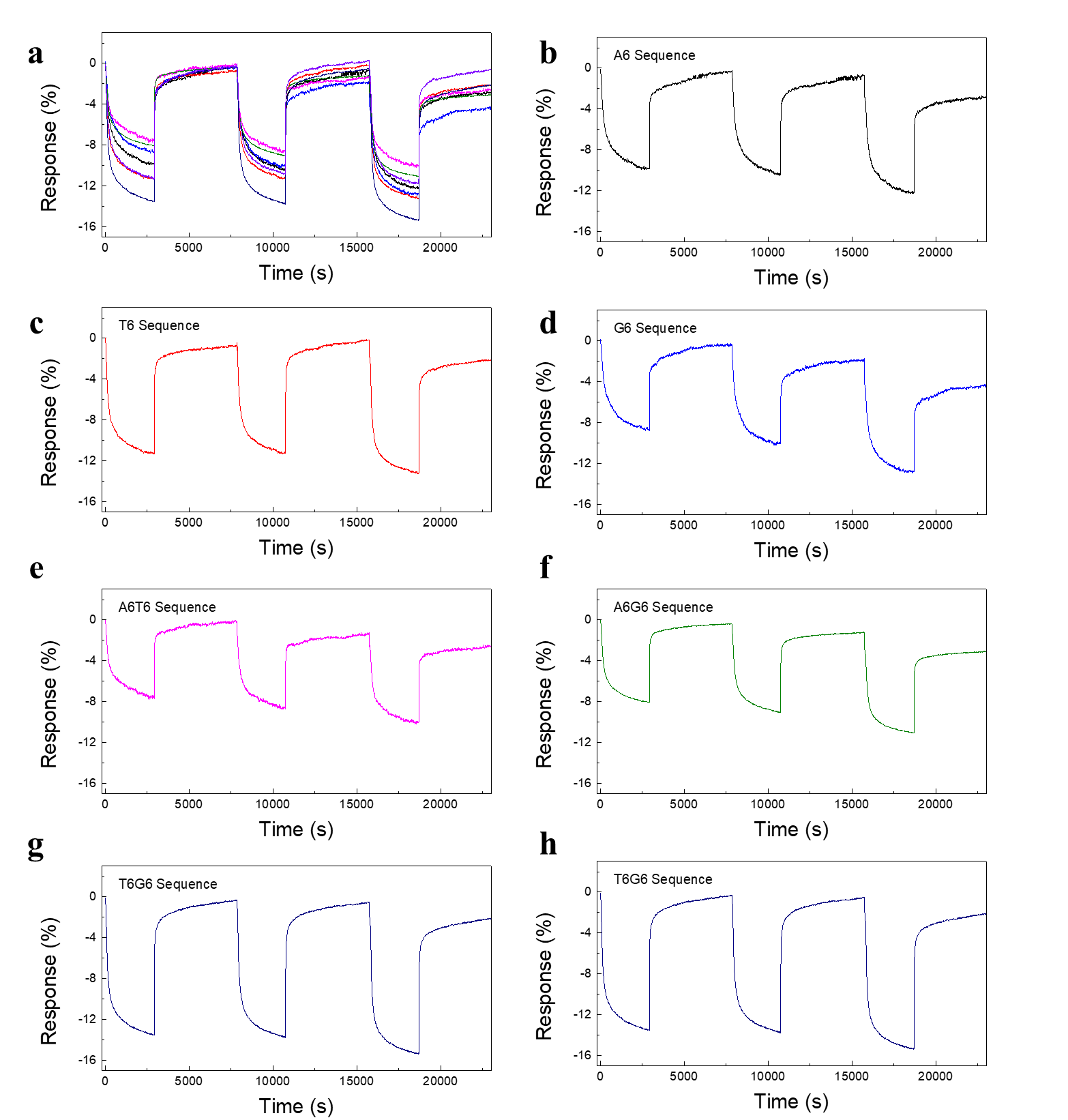


**Figure S9.** Response of NO_2_ individual gas under low-humidity condition.


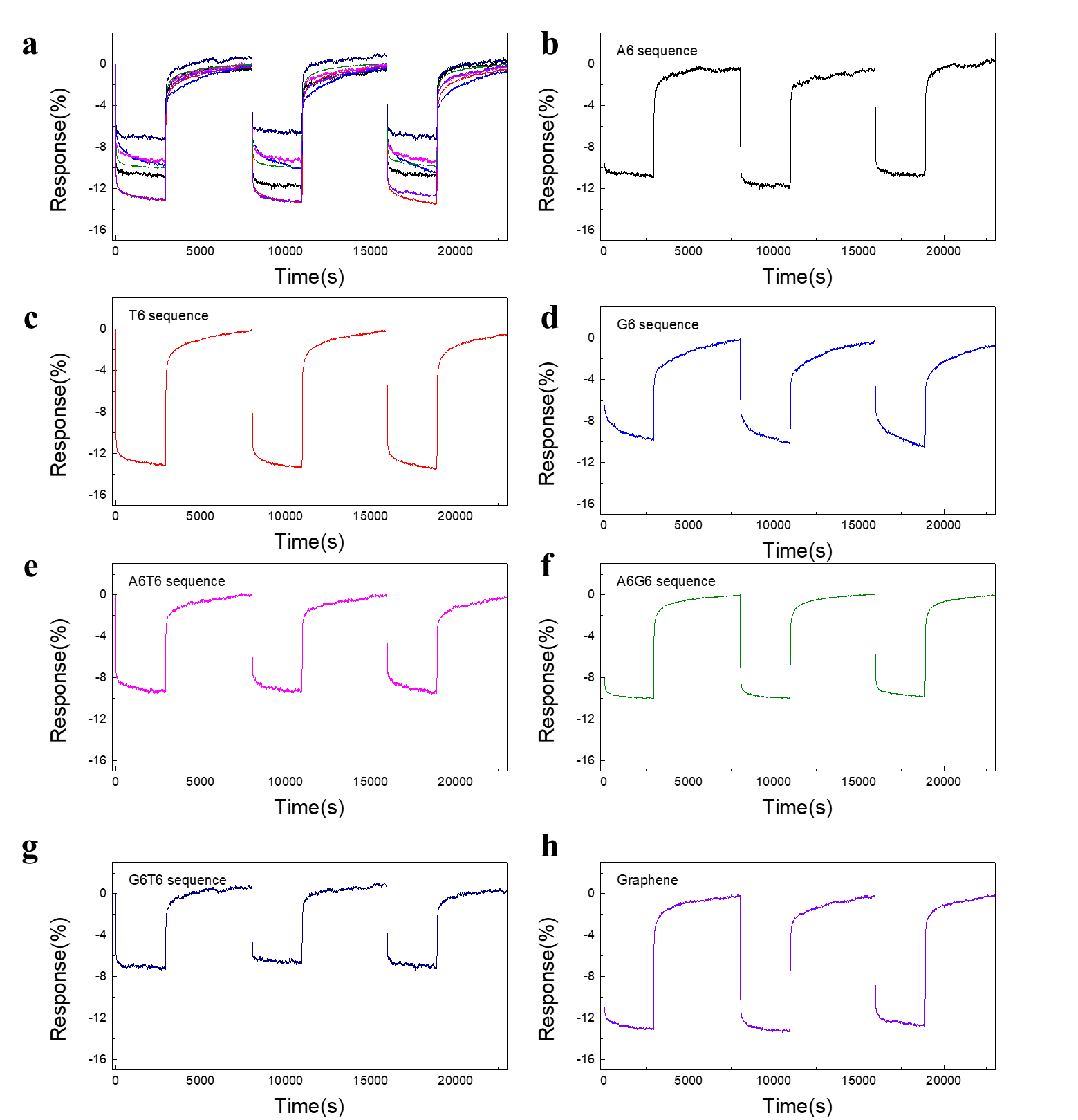


**Figure S10.** Response of individual NO gas under low-humidity condition.


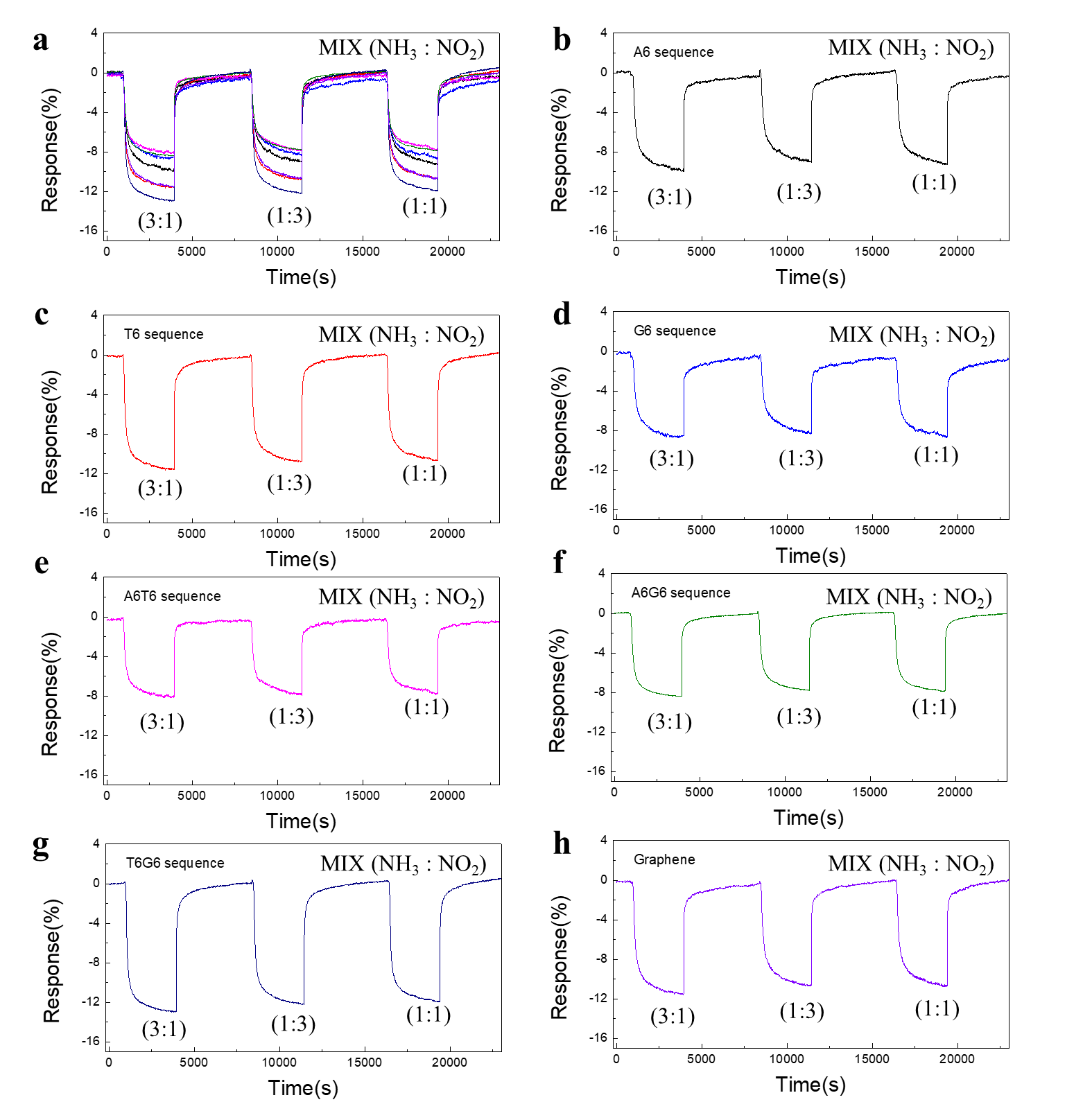


**Figure S11.** Response of NH_3_–NO_2_ mixed gases under low-humidity condition.


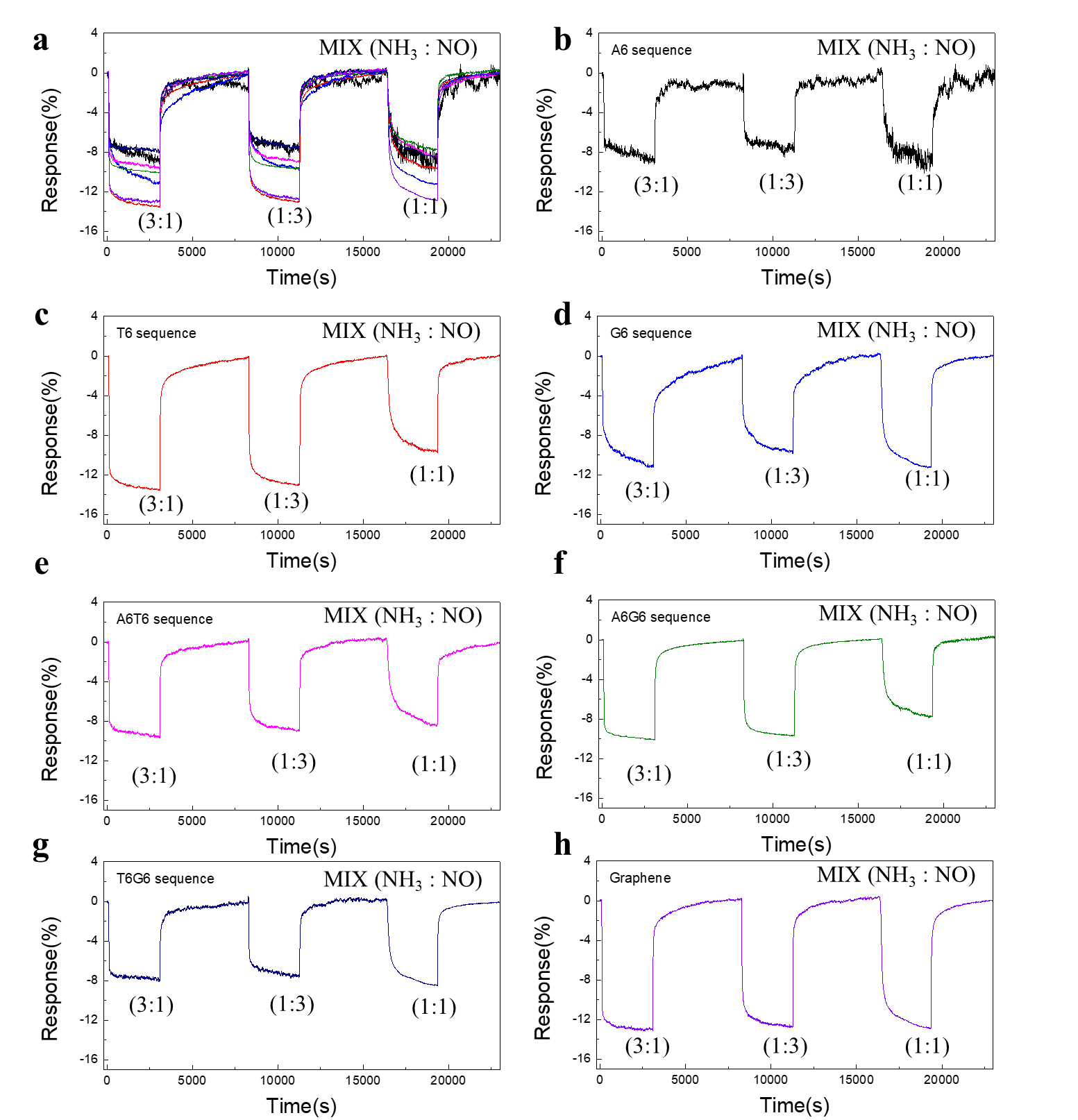


**Figure S12.** Response of NH_3_–NO mixed gases under low-humidity condition.


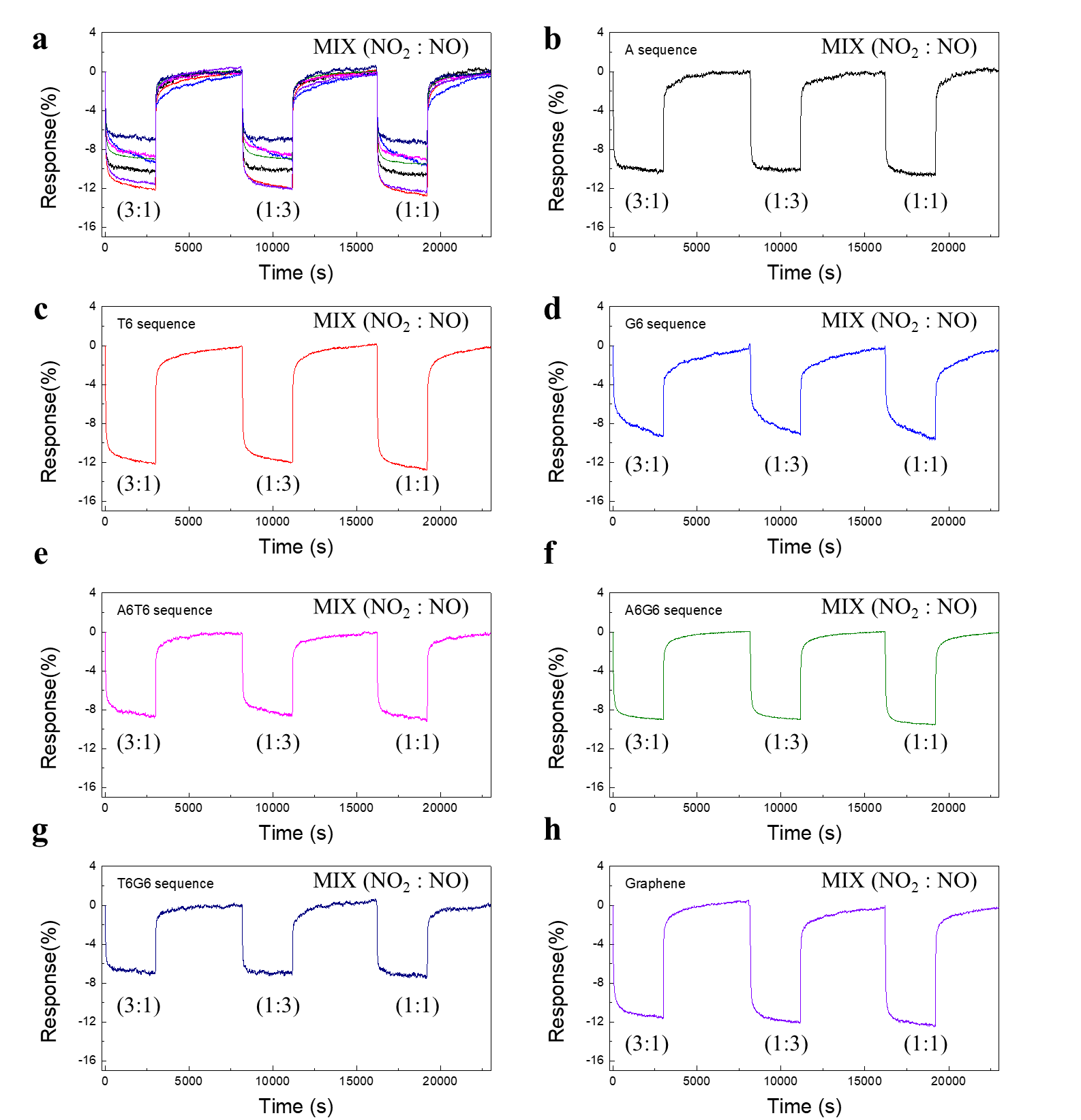


**Figure S13.** Response of NO_2_–NO mixed gases under low-humidity condition.


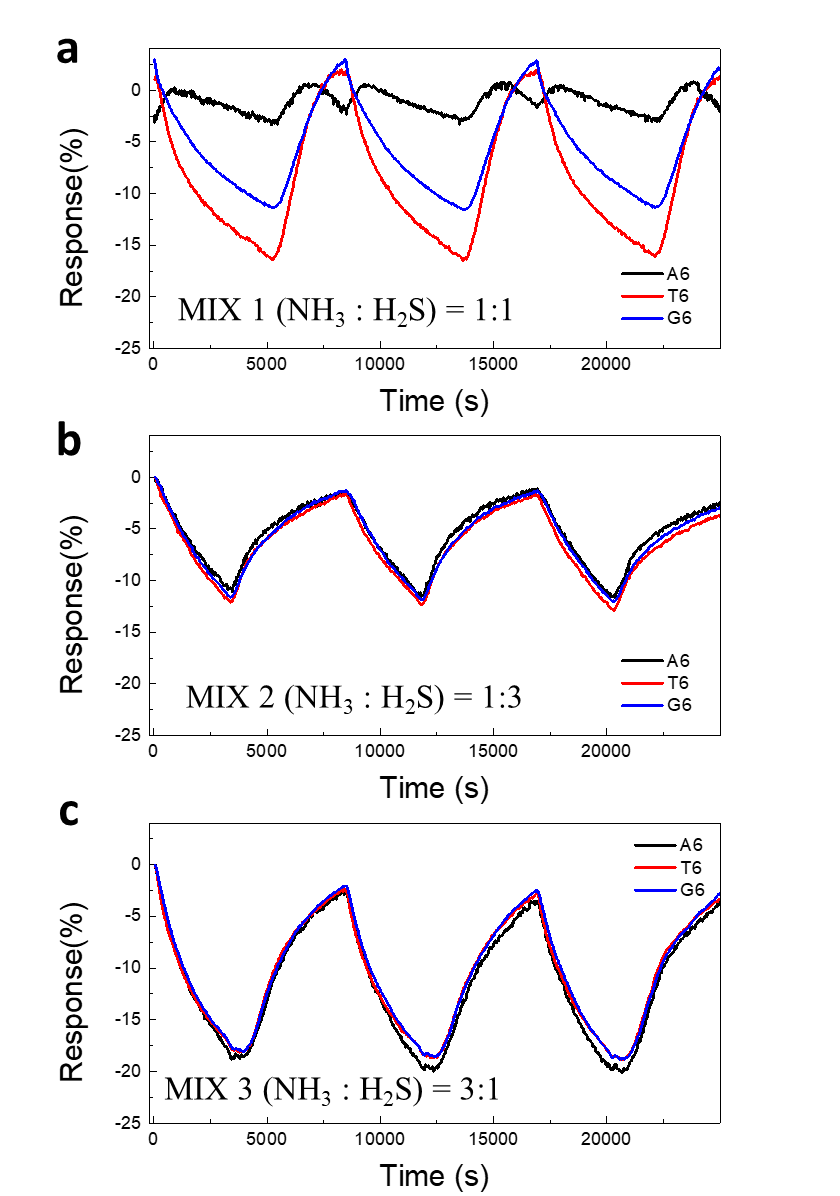


**Figure S14.** Response of NH_3_–H_2_S mixed gases under high-humidity condition.


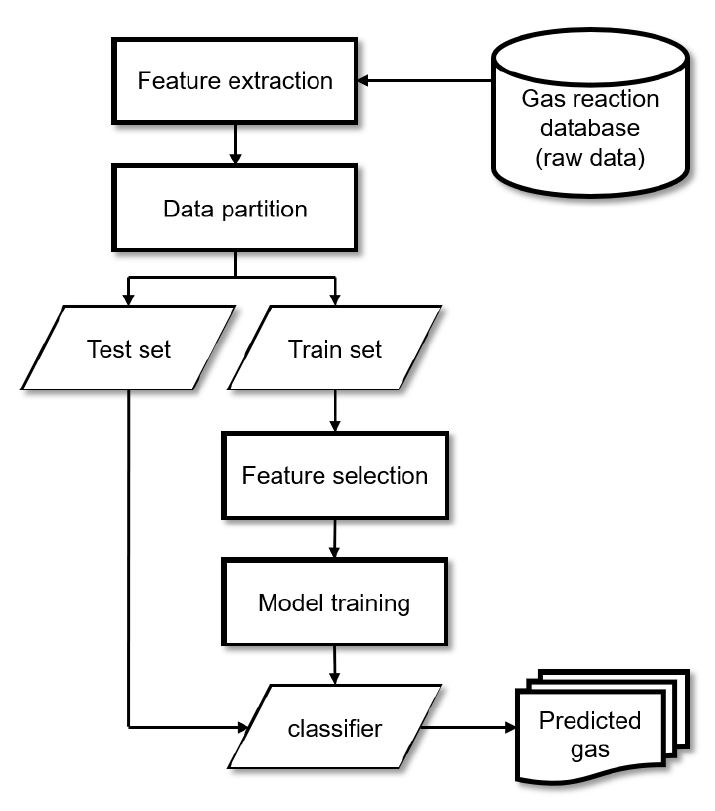


**Figure S15.** Feature selection analysis using Boruta algorithm.


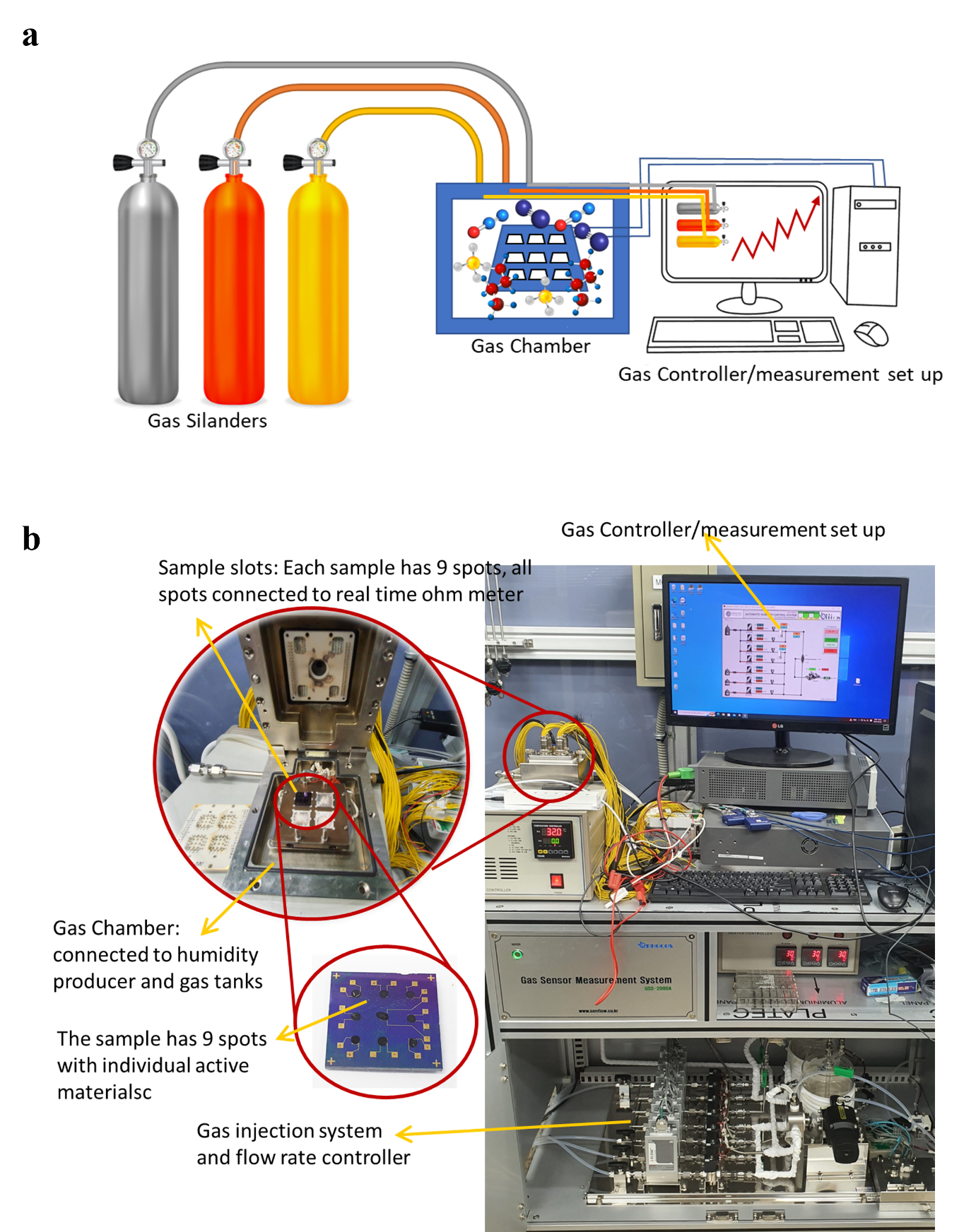


**Figure S16.** The schematics of experimental setup.

**Table S1.** Individual and mixture gases used for measurement.

|  | Individual gas | | |  | Mixture gas | | |
| --- | --- | --- | --- | --- | --- | --- | --- |
| Species | NO_2_ | NO | NH_3_ | Species | NO_2_/NO | NO_2_/NH_3_ | NO/NH_3_ |
| Concentration [ppm] | 2, 5, 10 | | | Mixing Ratio | 5:5, 2.5:7.5, 7.5:2.5 | | |

**Table S2.** Reference of gas sensor performance.

| Type of electrodes | Maximum Response | limit and range of detection | selectivity | Response time | Recovery time | Reference |
| --- | --- | --- | --- | --- | --- | --- |
| MDFG | 40% | 2 ppm | NO2, NO, and NH3 | 368 s | 114 s | This study |
| PANI-GO | - | - | NH3 | 150 s | 400 s | Javadian-Saraf ^1^ |
| PANI-MoS2-SnO2 | 1090% | 100 ppm | NH3 | 21 s | 130 s | A. Liu ^2^ |
| PANI-PVDF-G | 60% | 1 ppm | NH3 | 46 s | 198 s | Q. Wu ^3^ |
| PANI-PET:NH2-  MWCNTs | 117% | 50 ppm | NH3 | 47 s | - | Ma ^4^ |
| PPy-NiO | 65% | 45 ppm | NH3 | 12 s | 178 s | Thi Hien ^5^ |
| PANI-Nb2CTx | 65% | 10 ppm | NH3 | 218 s | 300 s | Wang ^6^ |
| PANI-h-NiO | 43% | 10 ppm | NH3 | 149 s | 257 s | Hu^7^ |
| PPy-G-Ag-Ag2O | - | - | NH3 | 60 s | 40 s | Shoeb ^8^ |
| PPy-SRGO | - | - | NH3 | 48 s | 234 s | Shahmoradi ^9^ |
| PANI-RGO | 620% | 100 ppm | NH3 | 219 s | 541 s | Luo ^10^ |
| LaNiMoSe2-GPANI | - | - | NH3 | 7~15 s | 6~35 s | Oh ^11^ |
| PANI-SnO2 | 200% | 7 ppm | NH3 | 44 s | 915 s | Feng ^12^ |
| PPy-GO-WO3 | 58% | 10 ppm | NH3 | 50 s | 120 s | Albaris & Karuppasamy ^13^ |
| PPy-V2O5-WO3 | 85% | 50 ppm | NH3 | 73 s | 101 s | Amarnath ^14^ |
| PANI-PVDFMWCNTs | 32% | 1 ppm | NH3 | 76 s | 26 s | T. Wu ^15^ |
| PANI-SrGe4O9 | 208% | 10 ppm | NH3 | 62 s | 223 s | Zhang ^16^ |
| PMMA-MoS2 | 54% | 500 ppm | NH3 | 10 s | 14 s | Abun ^17^ |
| PTh-MWCNTs | 88.7% | 2000 ppm | NH3 | 100 s | 90 s | Husain ^18^ |
| PANI-Ph-RGO | 773% | 100 ppm | NH3 | 300 s | 800 s | Tanguy ^19^ |
| PVDF-CeO2-GO | 1232% | 50 ppm | NH3 | 106 s | 11 s | Deshmukh ^20^ |
| ZnO-en-PPy | - | - | NH3 | 45 s | 55 s | Singh ^21^ |
| PANI-WO3 | 3400% | 100 ppm | NH3 | 30 s | 390 s | Fan ^22^ |
| PANI-GO-ZnO | - | 300 ppm | NH3 | 2 s | 164 s | Gaikwad ^23^ |
| PANI-WO3-CuCl2 | - | - | H2S | 10 s | - | Belkhamassa ^24^ |
| PBTTT-GO | 174% | 10 ppm | NO2 | 75 s | 523 s | Sahu ^25^ |
| PPy-SnO2 | 53.7% | 100 ppm | NO2 | 33 s | 881 s | Sakhare ^26^ |
| PPy-Fe2O3 | 221% | 10 ppm | NO2 | 150 s | 879 s | Wang ^27^ |
| PPy-pTSA-Ag | 68% | 100 ppm | NO2 | 148 s | 500 s | Karmakar ^28^ |
| porous laser-induced graphene (LIG) | 900% | 1 ppm | NO2 | 200 s | 700 s | Yang ^29^ |
| Pd/SnO2/G | 14.8 % | 2000 ppm | H2 | 34 s | 27 s | ^30^ |
| PPy/TiO2/G | 102.2% | 50 ppm | Aceton | 36 s | 16 s | ^31^ |
| PANI/ZnO/G | 25.6% | 5 ppm | Aceton | 22 s | 30 s | ^32^ |
| ZnO/G | 24% | 1 ppm | NH3 | 360 s | 180 s | ^33^ |
| SnO2/G | 17.7% | 100 ppm | ethanol | 9 s | 457 s | ^34^ |
| ZnO/G | 8% | 50 ppm | NO2 | 132 s | 164 s | ^35^ |

**Table S3.** Feature description

| Feature | Description | Number of features |
| --- | --- | --- |
| Magnitude | Down-sampled values of the magnitude  The maximum or minimum magnitude  Down-sampled values of the normalized magnitude | 31  1  31 |
| Derivative | Down-sampled values of derivative D  The maximum and minimum derivatives  The maximum and minimum second derivative during both injection and purging stages | 31  2  4 |
| Difference | The difference (D) in magnitude | 5 |
| Time constant | The time when the reaction reaches 30%, 60%, 90%, and 100% of its maximum or minimum value (and 90%, 60%, and 30% of its maximum or minimum value during the purging stage) | 7 |
| Area | The area under the eight intervals of the curve, where the interval is divided by the time constant features | 8 |

**Table S4.** Accuracy of classification model with low humidity condition.

| Low Humidity Condition | | Accuracy [%] | |
| --- | --- | --- | --- |
| Chemical Vapor Composition | Mixing ratio | With feature selection | With 1D CNN |
| NH_3_–NO_2_ | 1:1 | 96 | 100 |
|  | 1:3 | 100 | 100 |
|  | 3:1 | 100 | 100 |
| NH_3_–NO | 1:1 | 100 | 100 |
|  | 1:3 | 100 | 100 |
|  | 3:1 | 100 | 100 |
| NO_2_–NO | 1:1 | 100 | 100 |
|  | 1:3 | 99.5 | 100 |
|  | 3:1 | 100 | 100 |

**Table S5.** Performance of classification model with low humidity condition.

|  | With feature selection | | | | Using all features | | | |
| --- | --- | --- | --- | --- | --- | --- | --- | --- |
| Chemical Vapor Composition | Precision  [%] | Recall  [%] | F1 score  [%] | Compression ratio | Precision  [%] | Recall  [%] | F1 score  [%] | Compression ratio |
| NO_2_–NH_3_ | 98.67 | 98.67 | 98.69 | 7.01 | 98.14 | 98.08 | 98.11 | 1 |
| NO–NH_3_ | 100 | 100 | 100 | 4.62 | 100 | 100 | 100 | 1 |
| NO–NO_2_ | 99.33 | 99.83 | 99.85 | 6.02 | 99.48 | 99.47 | 99.47 | 1 |

**Table S6.** Accuracy of classification model with high humidity condition.

| High Humidity Condition | | Accuracy [%] | |
| --- | --- | --- | --- |
| Chemical Vapor Composition | Mixing ratio | With feature selection | With 1D CNN |
| NH_3_–H_2_S | NH_3_ | 85 | 100 |
|  | H_2_S | 96.5 | 100 |
|  | 1:1 | 100 | 100 |
|  | 1:3 | 100 | 100 |
|  | 3:1 | 100 | 100 |
| NH_3_–NO | NH_3_ | 100 | 100 |
|  | NO | 99.83 | 100 |
|  | 1:1 | 100 | 100 |
|  | 1:3 | 100 | 100 |
|  | 3:1 | 96.83 | 100 |
| H_2_S–NO | H_2_S | 100 | 100 |
|  | NO | 99 | 100 |
|  | 1:1 | 100 | 100 |
|  | 1:3 | 100 | 100 |
|  | 3:1 | 100 | 100 |

**Table S7.** Performance of classification model with high humidity condition.

|  | Classification Accuracy [%] | |
| --- | --- | --- |
| Chemical Vapor Composition | With feature selection | Using all features |
| NH_3_–H_2_S | 100 | 100 |
| NH_3_–NO | 98.94 | 97.23 |
| H_2_S–NO | 100 | 100 |

**Table S8.** Types of gases in human exhaled breath for lung and liver diseases.

| Disease | Type of Gas | Reference |
| --- | --- | --- |
| Lung Cancer | isobutene, methanol, ethanol, acetone, pentane, isoprene, isopropanol , dimethylsulfide, carbon disulphide, benzene, toluene | ^36^ |
| Liver cancer | 2,3-dihydro-benzofuran, methane-sulfonyl chloride, acetic acid, ethanol, hexanal, 1-octen-3-ol, octane, 3-Hydroxy-2-butanone, styrene, and decane  (set A: HCC patients/normal controls, B: cross-validation) | ^37-39^ |

**Table S9.** Existing commercialized exhaled gas analyzer product specifications.^40,41^

| Product Name | Exhaled Breath Analyzer | Exhaled Breath Analyzer |
| --- | --- | --- |
| Sampling Gas Volume | 10~15ml | 10~15ml |
| Measuring Time | 2min 30sec. (150sec.) | 2min 30sec. (150sec.) |
| Measuring Unit | ng/10ml, ppb | ppm |
| Sampling Method | Automatic suction | Automatic suction |
| Detectable Gases | Hydrogen, Hydrogen sulfide, Methyl mercaptan | Acetone, Isoprene, Other VOCs |

**References**

1 Javadian-Saraf, A., Hosseini, E., Wiltshire, B. D., Zarifi, M. H. & Arjmand, M. Graphene oxide/polyaniline-based microwave split-ring resonator: A versatile platform towards ammonia sensing. *Journal of Hazardous Materials* **418**, 126283 (2021).

2 Liu, A. *et al.* The gas sensor utilizing polyaniline/MoS2 nanosheets/SnO2 nanotubes for the room temperature detection of ammonia. *Sensors and Actuators B: Chemical* **332**, 129444 (2021).

3 Wu, Q. *et al.* An enhanced flexible room temperature ammonia gas sensor based on GP-PANI/PVDF multi-hierarchical nanocomposite film. *Sensors and Actuators B: Chemical* **334**, 129630 (2021).

4 Ma, J. *et al.* Multi-walled carbon nanotubes/polyaniline on the ethylenediamine modified polyethylene terephthalate fibers for a flexible room temperature ammonia gas sensor with high responses. *Sensors and Actuators B: Chemical* **334**, 129677 (2021).

5 Hien, H. T. *et al.* High NH3 sensing performance of NiO/PPy hybrid nanostructures. *Sensors and Actuators B: Chemical* **340**, 129986 (2021).

6 Wang, S. *et al.* Ultrathin Nb2CTx nanosheets-supported polyaniline nanocomposite: Enabling ultrasensitive NH3 detection. *Sensors and Actuators B: Chemical* **343**, 130069 (2021).

7 Hu, Q. *et al.* Design and preparation of hollow NiO sphere-polyaniline composite for NH3 gas sensing at room temperature. *Sensors and Actuators B: Chemical* **344**, 130179 (2021).

8 Shoeb, M., Mobin, M., Ahmad, S. & Naqvi, A. H. Facile synthesis of polypyrrole coated graphene Gr/Ag–Ag2O/PPy nanocomposites for a rapid and selective response towards ammonia sensing at room temperature. *Journal of Science: Advanced Materials and Devices* **6**, 223-233 (2021).

9 Shahmoradi, A., Hosseini, A., Akbarinejad, A. & Alizadeh, N. Noninvasive detection of ammonia in the breath of hemodialysis patients using a highly sensitive ammonia sensor based on a polypyrrole/sulfonated graphene nanocomposite. *Analytical Chemistry* **93**, 6706-6714 (2021).

10 Luo, G., Xie, L., He, M., Jaisutti, R. & Zhu, Z. Flexible fabric gas sensors based on reduced graphene-polyaniline nanocomposite for highly sensitive NH 3 detection at room temperature. *Nanotechnology* **32**, 305501 (2021).

11 Oh, W.-C. *et al.* Chemo-electrical gas sensors based on LaNiMoSe2 in graphene and conducting polymer PANI composite semiconductor nanocomposite. *Journal of Electronic Materials* **50**, 5754-5764 (2021).

12 Feng, Q., Zhang, H., Shi, Y., Yu, X. & Lan, G. Preparation and gas sensing properties of PANI/SnO2 hybrid material. *Polymers* **13**, 1360 (2021).

13 Albaris, H. & Karuppasamy, G. Investigation of NH3 gas sensing behavior of intercalated PPy–GO–WO3 hybrid nanocomposite at room temperature. *Materials Science and Engineering: B* **257**, 114558 (2020).

14 Amarnath, M., Heiner, A. & Gurunathan, K. Size controlled V2O5-WO3 nano-islands coated polypyrrole matrix: A unique nanocomposite for effective room temperature ammonia detection. *Sensors and Actuators A: Physical* **313**, 112211 (2020).

15 Wu, T., Lv, D., Shen, W., Song, W. & Tan, R. Trace-level ammonia detection at room temperature based on porous flexible polyaniline/polyvinylidene fluoride sensing film with carbon nanotube additives. *Sensors and Actuators B: Chemical* **316**, 128198 (2020).

16 Zhang, J., Wu, C., Li, T., Xie, C. & Zeng, D. Highly sensitive and ultralow detection limit of room-temperature NO2 sensors using in-situ growth of PPy on mesoporous NiO nanosheets. *Organic Electronics* **77**, 105504 (2020).

17 Abun, A., Huang, B.-R., Saravanan, A., Kathiravan, D. & Hong, P.-D. Effect of PMMA on the surface of exfoliated MoS2 nanosheets and their highly enhanced ammonia gas sensing properties at room temperature. *Journal of Alloys and Compounds* **832**, 155005 (2020).

18 Husain, A., Ahmad, S. & Mohammad, F. Electrical conductivity and ammonia sensing studies on polythiophene/MWCNTs nanocomposites. *Materialia* **14**, 100868 (2020).

19 Tanguy, N. R., Wiltshire, B., Arjmand, M., Zarifi, M. H. & Yan, N. Highly sensitive and contactless ammonia detection based on nanocomposites of phosphate-functionalized reduced graphene oxide/polyaniline immobilized on microstrip resonators. *ACS applied materials & interfaces* **12**, 9746-9754 (2020).

20 Deshmukh, K. & Pasha, S. K. Room temperature ammonia sensing based on graphene oxide integrated flexible polyvinylidenefluoride/cerium oxide nanocomposite films. *Polymer-Plastics Technology and Materials* **59**, 1429-1446 (2020).

21 Singh, P., Kushwaha, C. S., Singh, V. K., Dubey, G. & Shukla, S. K. Chemiresistive sensing of volatile ammonia over zinc oxide encapsulated polypyrrole based nanocomposite. *Sensors and Actuators B: Chemical* **342**, 130042 (2021).

22 Fan, G. *et al.* Enhanced room-temperature ammonia-sensing properties of polyaniline-modified WO3 nanoplates derived via ultrasonic spray process. *Sensors and Actuators B: Chemical* **312**, 127892 (2020).

23 Gaikwad, G., Patil, P., Patil, D. & Naik, J. Synthesis and evaluation of gas sensing properties of PANI based graphene oxide nanocomposites. *Materials Science and Engineering: B* **218**, 14-22 (2017).

24 Belkhamssa, N., Ksibi, M., Shih, A. & Izquierdo, R. Fabrication of Fast Responsive and Insensitive-Humidity Sensor Based on Polyaniline-WO 3-CuCl 2 for Hydrogen Sulfide Detection. *IEEE Sensors Journal* **21**, 9716-9722 (2020).

25 Sahu, P. K., Pandey, R. K., Dwivedi, R., Mishra, V. & Prakash, R. Polymer/Graphene oxide nanocomposite thin film for NO2 sensor: An in situ investigation of electronic, morphological, structural, and spectroscopic properties. *Scientific reports* **10**, 1-13 (2020).

26 Sakhare, R., Navale, Y., Jadhav, Y., Mulik, R. & Patil, V. in *Techno-Societal 2020* 1021-1029 (Springer, 2021).

27 Wang, C. *et al.* One-step synthesis of polypyrrole/Fe2O3 nanocomposite and the enhanced response of NO2 at low temperature. *Journal of colloid and interface science* **560**, 312-320 (2020).

28 Karmakar, N. *et al.* Room temperature NO2 gas sensing properties of p-toluenesulfonic acid doped silver-polypyrrole nanocomposite. *Sensors and Actuators B: Chemical* **242**, 118-126 (2017).

29 Yang, L. *et al.* Novel gas sensing platform based on a stretchable laser-induced graphene pattern with self-heating capabilities. *Journal of materials chemistry A* **8**, 6487-6500 (2020).

30 Dhall, S., Kumar, M., Bhatnagar, M. & Mehta, B. Dual gas sensing properties of graphene-Pd/SnO2 composites for H2 and ethanol: Role of nanoparticles-graphene interface. *International Journal of Hydrogen Energy* **43**, 17921-17927 (2018).

31 Xiang, C. *et al.* Ammonia sensor based on polypyrrole–graphene nanocomposite decorated with titania nanoparticles. *Ceramics International* **41**, 6432-6438 (2015).

32 Zhang, D., Wu, Z. & Zong, X. Metal-organic frameworks-derived zinc oxide nanopolyhedra/S, N: graphene quantum dots/polyaniline ternary nanohybrid for high-performance acetone sensing. *Sensors and Actuators B: Chemical* **288**, 232-242 (2019).

33 Chen, Z.-W., Hong, Y.-Y., Lin, Z.-D., Liu, L.-M. & Zhang, X.-W. Enhanced formaldehyde gas sensing properties of ZnO nanosheets modified with graphene. *Electronic Materials Letters* **13**, 270-276 (2017).

34 Zhao, C. *et al.* Facile synthesis of SnO2 hierarchical porous nanosheets from graphene oxide sacrificial scaffolds for high-performance gas sensors. *Sensors and Actuators B: Chemical* **258**, 492-500 (2018).

35 Liu, X., Sun, J. & Zhang, X. Novel 3D graphene aerogel–ZnO composites as efficient detection for NO2 at room temperature. *Sensors and Actuators B: Chemical* **211**, 220-226 (2015).

36 Machado, R. F. *et al.* Detection of lung cancer by sensor array analyses of exhaled breath. *American journal of respiratory and critical care medicine* **171**, 1286-1291 (2005).

37 Amal, H. *et al.* The scent fingerprint of hepatocarcinoma: in-vitro metastasis prediction with volatile organic compounds (VOCs). *International journal of nanomedicine* **7**, 4135 (2012).

38 Xue, R. *et al.* Investigation of volatile biomarkers in liver cancer blood using solid‐phase microextraction and gas chromatography/mass spectrometry. *Rapid Communications in Mass Spectrometry: An International Journal Devoted to the Rapid Dissemination of Up‐to‐the‐Minute Research in Mass Spectrometry* **22**, 1181-1186 (2008).

39 Qin, T. *et al.* The screening of volatile markers for hepatocellular carcinoma. *Cancer epidemiology, biomarkers & prevention* **19**, 2247-2253 (2010).

40 Marzorati, D. *et al.* MOS sensors array for the discrimination of lung cancer and at-risk subjects with exhaled breath analysis. *Chemosensors* **9**, 209 (2021).

41 iSenLab. *Twin Breasor ll*. (2019). at <<http://www.isenlab.com/page/product1.php>>
